# Supplementary material for: Who supports science-related populism? A nationally representative survey on the prevalence and explanatory factors of populist attitudes toward science in Switzerland
Source: PLoS One. 2022 Aug 8;17(8):e0271204. doi: 10.1371/journal.pone.0271204 (PMC9359586; doi:10.1371/journal.pone.0271204)
Supplement: S1 Appendix. Additional information, tables, and figures — (PDF) [file pone.0271204.s001.pdf]

S1 Appendix

for

Mede, N. G., Schäfer, M. S., Metag, J., & Klinger, K. (2022). Who supports science-related populism? A nationally representative survey on the prevalence and explanatory factors of populist attitudes toward science in Switzerland. *PloS One*. https://doi.org/10.1371/journal.pone.0271204

Contents

Introduction 3

Data and Code for Reproducing the Analyses 3

Data Handling and Important R Packages 3

Assumption Checks for the Regression Models 3

Quantifying Science-Related Populist Attitudes: The SciPop Score 4

Sensitivity Tests With Trimmed Survey Weights 5

Supplementary Tables 6

A1. Overview of variables used in the analyses 6

A2. Ms and SDs of science-related populist attitudes, its components, and SciPop Scale items 9

A3. One-sample t-tests for dimensions of science-related populist attitudes 10

A4. Stepwise multiple linear regressions (DV: science-related populist attitudes) 11

A5. Stepwise multiple linear regressions (DV: conceptions of the ordinary people) 12

A6. Stepwise multiple linear regressions (DV: conceptions of the academic elite) 13

A7. Stepwise multiple linear regressions (DV: demands for decision-making sovereignty) 14

A8. Stepwise multiple linear regressions (DV: demands for truth-speaking sovereignty) 15

A9. Quadratic multiple linear regressions (DV: science-related populist attitudes) 16

A10. Multiple linear regressions explaining science-related populist attitudes (sensitivity test 1: Bollenian instead of Goertzian SciPop Scores) 17

A11. Multiple linear regressions explaining science-related populist attitudes and its components (sensitivity test 2: trimmed instead of original weights) 18

Supplementary Figures 19

A1. Relative response frequencies of SciPop Scale items 19

A2. Mean SciPop Scores in sample subgroups 20

A3. Means of subscale scores in sample subgroups 21

A4. Plot of two-lines test of relationship between SciPop Score and political orientation 22

References 23

Introduction

In this document we provide information, tables, and figures supplementing the analyses presented in our article "Who supports science-related populism? A nationally representative survey on the prevalence and explanatory factors of populist attitudes toward science in Switzerland," which has been published in *PLOS One*.

Data and Code for Reproducing the Analyses

All analyses were conducted in R (version 4.1.0). We share the R syntax we used for the analyses at https://osf.io/qj4xr/. Survey data and additional materials (e.g., the questionnaires and a methodological report, the former in German, French, and Italian, the latter only in German) can be accessed/requested in the online repository *SWISSUbase* (doi: 10.23662/FORS-DS-1229-1).

Data Handling and Important R Packages

The analyses relied on weighted survey data, which were handled with the R package survey v4.1-1 [1]. For the two-lines test (see Figure A4), we used code provided by Uri Simonsohn, who developed the test [2].

Assumption Checks for the Regression Models

To test assumptions for multiple linear regressions we examined distributions and covariances for all regression models (see Table 2 and Tables A4-A9). These tests can be reproduced with the R syntax. They neither suggest multicollinearity of explanatory variables nor non-normality or heteroskedasticity of the residuals of any of the regression models [3,4].

Quantifying Science-Related Populist Attitudes: The SciPop Score

To obtain a single aggregate score which quantifies propensity and aversion to science-related populism, we followed the "Goertz approach" [5]: We computed mean values of each of the four 2-item subscales for every respondent and determined the smallest of these four values to represent their "SciPop Score." The four subscale mean values attained during this procedure were used to quantify endorsement of the four components of science-related populist attitudes, i.e. *conceptions of the ordinary people*, *conceptions of the academic elite*, *demands for decision-making sovereignty*, and *demands for truth-speaking sovereignty*. Higher SciPop Scores and higher subscale scores indicate stronger support for science-related populism and its components.

Averaging participants' responses to the SciPop Scale items would have been another way to compose an aggregate score for science-related populist attitudes, a procedure Wuttke et al. [5] described as "Bollen approach." We decided against this approach, because it would produce scores which are not in line with the conceptual premise that science-related populism is a non-compensatory concept, i.e. that it relies on the concurrent presence of all its four components [6]. For example, Bollenian scores (means of all scale items) would indicate similar degrees of science-related populism for respondent A, who endorses some of its components fully but rejects others completely, and for respondent B who endorses all components moderately—although only respondents like B can be understood as supporters of science-related populism because all its facets are concurrently present in them. Goertzian scores (minimum subscale means) would not indicate similar populism degrees for A and B, however. They would be smaller in A's case, where one component is absent, and bigger in B's case, where all components are present. Accordingly, the Goertz approach can usefully account for the concurrency criterion of non-compensatory phenomena like science-related populism, and, as such, represents a useful analytical procedure for us to translate responses to the SciPop Scale into a single numerical value indicating science-related populist attitudes. This approach corresponds with recent research on political populism, which

increasingly employs Goertzian instead of Bollenian aggregation procedures [7–9]. Because the Bollen approach is still frequently used in populism research, we nevertheless ran additional analyses testing whether we would have obtained different results if we had not applied the Goertz but the Bollen approach to compute the SciPop Score. Accordingly, we repeated all hypothesis tests using Bollenian scores (mean of all SciPop Scale items) instead of Goertzian scores (minimum subscale mean).

Sensitivity Tests With Trimmed Survey Weights

We tested if our analyses were sensitive to variance in survey weights, because extreme variance can introduce bias into regression analyses [10]. The weights in our data do not have high variance (SD = 1.44) and ca. 95% are between 7^{-1} and 7, so we did not expect such bias to be substantial. However, the survey data contained a few very small or large weights (range = 0.04 to 23.56). One way to control for these outliers is to constrain the range of survey weights in a process called trimming [11]. When specifying this range, we decided against general rules of thumb that consider all weights smaller than 7^{-1} and bigger than as 7 extreme, for example [12]. Instead, we used a sample-specific trimming range, which accounts for the characteristic variance of weights in our data set and can therefore be considered a more reliable approach to weight trimming [13].

To define that trimming range, we applied the interquartile-range (IQR) method, which is frequently employed in public opinion research [13–15]. It uses the median of weights (*Me*) and the spread difference between the 25th and 75th percentiles of the weights (*IQR*) to determine all weights outside the interval between $Me_{\text{weights}} - (k \times IQR_{\text{weights}})^{-1}$ and $Me_{\text{weights}} + k \times IQR_{\text{weights}}$ as extreme weights. For k, we chose $k = 6$, which was also used by Chowdhury et al. [14], for example. With this procedure, we classified all weights smaller than 4.56^{-1} (= 0.22) and bigger than 4.56 as extreme. We then fed these cut-off values into a recursive trimming function to force weights to be within this interval [15], and repeated the hypothesis tests, whose results are presented in Table A11.

Supplementary Tables

Table A1. Overview of variables used in the analyses

Variable	Type	Items	Levels in analyses
Science-related populist attitudes	Composite score (Goertz approach)	[SciPop Scale, see items below]	continuous, higher values indicate stronger endorsement of science-related populist attitudes (possible range: 1 to 5)
Conceptions of the ordinary people	Mean score (unweighted average)	What unites the ordinary people is that they trust their common sense in everyday life.	1 = do not agree at all, 5 = agree completely
		Ordinary people are of good and honest character.	1 = do not agree at all, 5 = agree completely
Conceptions of the academic elite	Mean score (unweighted average)	Scientists are only after their own advantage.	1 = do not agree at all, 5 = agree completely
		Scientists are in cahoots with politics and business.	1 = do not agree at all, 5 = agree completely
Demands for decision-making sovereignty	Mean score (unweighted average)	The people should have influence on the work of scientists.	1 = do not agree at all, 5 = agree completely
		People like me should be involved in decisions about the topics scientists research.	1 = do not agree at all, 5 = agree completely
Demands for truth-speaking sovereignty	Mean score (unweighted average)	In case of doubt, one should rather trust the life experience of ordinary people than the estimations of scientists.	1 = do not agree at all, 5 = agree completely
		We should rely more on common sense and less on scientific studies.	1 = do not agree at all, 5 = agree completely
Age	Single item (continuous)	In which year were you born?	[Age in years]
Gender	Single item (categorical)	[Determined by interviewer]	0 = male, 1 = female

Education	Single item (dummy-coded)	What is your educational background?	1 = compulsory school, 2 = secondary education, 3 = university degree
Proximity to science	Composite score[a]	Are you a scientist yourself?	0 = no, 1 = yes
		Do you know a scientist personally?	0 = no, 1 = yes
		Are you professionally involved with science?	0 = no, 1 = yes
		Do you have family members who have studied or are studying?	0 = no, 1 = yes
Urbanity of residence	Single item (continuous)[b]	[Log-transformed inhabitant counts of residence municipalities, which were inferred from postal codes]	continuous, higher values indicate higher urbanity
Swiss region	Single item (dummy-coded)	[Inferred from postal codes]	1 = German-speaking, 2 = French-speaking, 3 = Italian-speaking
Political orientation	Single item (continuous)	How would you classify your own political orientation?	1 = very left-leaning, 7 = very right-leaning
Religiosity	Single item (continuous)	How religious would you consider yourself?	1 = not at all religious, 5 = very religious
Interest in science	Single item (continuous)	How interested are you in science and research?	1 = not interested at all, 5 = very strongly interested
Scientific literacy	Composite score[c]	The continents on which we live have been moving for millions of years. (correct)	1 = certainly wrong, 2 = rather wrong, 3 = rather true, 4 = certainly true, 98 = don’t know
		Electrons are smaller than atoms. (correct)	1 = certainly wrong, 2 = rather wrong, 3 = rather true, 4 = certainly true, 98 = don’t know
		Antibiotics kill viruses as well as bacteria. (false)	1 = certainly wrong, 2 = rather wrong, 3 = rather true, 4 = certainly true, 98 = don’t know
		The genes of the mother decide if the child will be a boy or a girl. (false)	1 = certainly wrong, 2 = rather wrong, 3 = rather true, 4 = certainly true, 98 = don’t know
		Scientific theories never change. (false)	1 = certainly wrong, 2 = rather wrong, 3 = rather true, 4 = certainly true, 98 = don’t know
Trust in science	Single item (continuous)	How high is your trust in science in general?	1 = very low, 5 = very high

Trust in scientists	Single item (continuous)	How high is your trust university scientists?	1 = very low, 5 = very high

Note. Original items were in German, French, and Italian, but we translated them into English for this article.

[a] Respondents who reported working as scientists were assigned a 4. Respondents were assigned a 3 if they answered "yes" to all three remaining questions; a 2 if they answered "yes" to two of these questions; a 1 if they answered "yes" to one of these questions; a 0 if they answered "yes" to none of these questions. The resulting score thus ranged from 0 (least proximity to science) to 4 (greatest proximity to science).

[b] Inferred from log-transformed inhabitant counts of respondents' residence municipalities, which were obtained by merging their postal codes with Swiss census data. Log-transformation was advisable as inhabitant counts were approximately log-normally distributed in the survey data. This pattern corresponds with previous analyses of Swiss census data showing that inhabitant counts of Swiss municipalities follow Zipf's Law [16].

[c] Respondents were given -2 points for every correct (false) statement they were certain it was wrong (true), -1 point for every correct (false) statement they were rather sure it was wrong (true), 0 points for every item for which they did not know if it was wrong or true, 1 point for every correct (false) statement they were rather sure it was true (wrong), and 2 points for every correct (wrong) statement they were certain it was true (wrong). The composite score was calculated by summing up all points.

Table A2. Means and standard deviations of science-related populist attitudes, its components, and SciPop Scale items in population groups

	Total *M* (*SD*)	Age (years) *M* (*SD*)				Gender *M* (*SD*)		Education *M* (*SD*)		
		15-29	30-44	45-59	60+	Male	Female	Compulsory school	Secondary education	University degree
Science-related populist attitudes	2.22 (0.80)	2.02 (0.80)	2.38 (0.87)	2.23 (0.79)	2.32 (0.73)	2.16 (0.81)	2.29 (0.78)	2.13 (0.82)	2.41 (0.81)	2.06 (0.72)
Conceptions of the ordinary people	3.29 (0.92)	3.20 (0.84)	3.31 (0.88)	3.21 (0.97)	3.51 (0.89)	3.23 (0.97)	3.35 (0.85)	3.53 (0.78)	3.39 (0.88)	2.99 (0.97)
What unites the ordinary people is that they trust their common sense in everyday life.	3.46 (1.03)	3.34 (0.94)	3.45 (0.99)	3.45 (1.09)	3.60 (1.05)	3.36 (1.08)	3.55 (0.98)	3.61 (0.97)	3.59 (0.98)	3.18 (1.09)
Ordinary people are of good and honest character.	3.12 (1.10)	3.04 (1.16)	3.19 (0.98)	2.97 (1.12)	3.43 (1.01)	3.09 (1.15)	3.16 (1.05)	3.44 (1.00)	3.19 (1.06)	2.81 (1.13)
Conceptions of the academic elite	2.77 (0.88)	2.49 (0.88)	2.86 (0.81)	2.87 (0.89)	2.85 (0.85)	2.74 (0.91)	2.80 (0.85)	2.68 (0.88)	2.89 (0.87)	2.70 (0.87)
Scientists are only after their own advantage.	2.61 (1.03)	2.37 (0.99)	2.49 (1.01)	2.63 (1.01)	2.89 (1.04)	2.60 (1.05)	2.62 (1.00)	2.52 (0.96)	2.71 (1.08)	2.55 (1.00)
Scientists are in cahoots with politics and business.	2.93 (1.08)	2.61 (1.07)	3.23 (1.00)	3.10 (1.08)	2.80 (1.03)	2.88 (1.10)	2.98 (1.06)	2.84 (1.07)	3.07 (1.09)	2.83 (1.06)
Demands for decision-making sovereignty	2.87 (0.97)	3.02 (0.97)	2.93 (1.00)	2.78 (0.99)	2.80 (0.89)	2.84 (1.00)	2.89 (0.93)	2.92 (1.07)	3.00 (0.93)	2.67 (0.92)
The people should have influence on the work of scientists.	2.86 (1.13)	3.04 (1.07)	2.65 (1.18)	2.81 (1.11)	2.89 (1.18)	2.80 (1.19)	2.91 (1.07)	2.81 (1.18)	3.08 (1.12)	2.61 (1.06)
People like me should be involved in decisions about the topics scientists research.	2.88 (1.16)	3.04 (1.17)	3.21 (1.18)	2.75 (1.15)	2.71 (1.09)	2.88 (1.18)	2.87 (1.14)	3.05 (1.23)	2.91 (1.16)	2.71 (1.09)
Demands for truth-speaking sovereignty	3.16 (0.96)	2.73 (0.98)	3.26 (0.88)	3.23 (0.89)	3.40 (0.98)	3.05 (1.03)	3.26 (0.89)	3.07 (0.95)	3.43 (0.90)	2.86 (0.96)
In case of doubt. one should rather trust the life experience of ordinary people than the estimations of scientists.	3.10 (1.02)	2.84 (1.03)	3.15 (0.81)	3.16 (1.04)	3.27 (1.04)	3.06 (1.10)	3.15 (0.93)	3.07 (0.87)	3.34 (0.99)	2.82 (1.07)
We should rely more on common sense and less on scientific studies.	3.21 (1.18)	2.63 (1.14)	3.38 (1.26)	3.31 (1.04)	3.52 (1.20)	3.04 (1.20)	3.37 (1.14)	3.07 (1.35)	3.52 (1.08)	2.89 (1.09)

Note: *N* between 1,036 and 1,045. Values based on survey weights (computed with the R package survey v4.1-1).

Table A3. One-sample t-tests for dimensions of science-related populist attitudes

	Conceptions of the ordinary people			Conceptions of the academic elite			Demands for decision-making sovereignty			Demands for truth-speaking sovereignty		
Dimension	ΔM	*t (df)*	α	ΔM	*t (df)*	α	ΔM	*t (df)*	α	ΔM	*t (df)*	α
Conceptions of the ordinary people	-	-										
Conceptions of the academic elite	0.52	9.24 (1,048)	< 0.001	-	-							
Demands for decision-making sovereignty	0.43	7.36 (1,048)	< 0.001	-0.09	-1.67 (1,048)	0.577	-	-				
Demands for truth-speaking sovereignty	0.13	2.39 (1,048)	0.101	-0.39	-7.84 (1,048)	< 0.001	-4.96	-0.29 (1,048)	< 0.001	-	-	

Note. T-tests were run with survey weights (using the R package survey v4.1-1) and were based on Bonferroni-corrected p values with $p_i = \frac{\alpha}{m}$, where p is the corrected significance level, α is the desired overall significance level, and m is the number of hypothesis tests. The number of hypothesis tests was m = 6, hence p = 0.00017 for α = 0.001, p = 0.00167 for α = 0.01, and p = 0.00833 for α = 0.05.

Table A4. Stepwise multiple linear regressions (DV: science-related populist attitudes)

	Step 1: Sociodemographic variables			Step 2: Political orientation and religiosity			Step 3: General perceptions of science		
Explanatory Variable	*b (SE)*	β	*p*	*b (SE)*	β	*p*	*b (SE)*	β	*p*
Intercept	2.72 (0.36)	2.21	< 0.001	2.26 (0.36)	2.21	< 0.001	2.89 (0.42)	2.21	< 0.001
Age	0.00 (0.00)	0.11	0.226	0.00 (0.00)	0.07	0.478	0.00 (0.00)	0.00	0.967
Gender (0 = male, 1 = female)	0.04 (0.09)	0.04	0.652	0.04 (0.09)	0.04	0.639	0.07 (0.08)	0.07	0.441
Education (ref. secondary education)									
Compulsory school	-0.35 (0.16)	-0.35	0.037	-0.37 (0.15)	-0.37	0.012	-0.37 (0.15)	-0.37	0.012
University degree	-0.22 (0.09)	-0.22	0.011	-0.21 (0.08)	-0.21	0.013	-0.16 (0.08)	-0.16	0.042
Proximity to science	-0.15 (0.04)	-0.36	< 0.001	-0.13 (0.04)	-0.32	< 0.001	-0.11 (0.04)	-0.26	0.002
Urbanity of residence	-0.01 (0.03)	-0.03	0.755	0.00 (0.03)	-0.01	0.925	-0.01 (0.03)	-0.03	0.713
Swiss region (ref. French-speaking)									
German-speaking	-0.24 (0.14)	-0.24	0.087	-0.22 (0.11)	-0.22	0.044	-0.17 (0.11)	-0.17	0.106
Italian-speaking	-0.32 (0.16)	-0.32	0.044	-0.30 (0.13)	-0.30	0.021	-0.24 (0.12)	-0.24	0.044
Political orientation (1 = left, 7 = right)				0.09 (0.05)	0.23	0.063	0.09 (0.05)	0.24	0.052
Religiosity				0.04 (0.04)	0.10	0.307	0.02 (0.04)	0.04	0.626
Interest in science and research							0.09 (0.03)	0.18	0.013
Scientific literacy							-0.04 (0.01)	-0.27	< 0.001
Trust in science							-0.05 (0.08)	-0.08	0.501
Trust in scientists							-0.12 (0.07)	-0.19	0.098
Adj. R²		0.10			0.13			0.18	
F change (df)		—			4.03 (2, 904)	0.018		8.72 (4, 900)	< 0.001
AIC		2504.27			2482.49			2434.00	

Note: *N* = 915. Regressions were run with survey weights using the R package survey v4.1-1. Standardization of *b* estimates follows Gelman's [17] suggestion to rescale the estimates by dividing them by two standard deviations instead of one. Assumption checks, which can be reproduced with the R syntax, neither suggest multicollinearity of explanatory variables nor non-normality or heteroskedasticity of the residuals of any of the regression models.

Table A5. Stepwise multiple linear regressions (DV: conceptions of the ordinary people)

	Step 1: Sociodemographic variables			Step 2: Political orientation and religiosity			Step 3: General perceptions of science		
Explanatory Variable	*b (SE)*	β	*p*	*b (SE)*	β	*p*	*b (SE)*	β	*p*
Intercept	3.99 (0.31)	3.29	< 0.001	3.68 (0.33)	3.29	< 0.001	3.78 (0.38)	3.29	< 0.001
Age	0.01 (0.00)	0.36	< 0.001	0.01 (0.00)	0.33	0.001	0.01 (0.00)	0.29	0.002
Gender (0 = male, 1 = female)	-0.08 (0.09)	-0.08	0.338	-0.08 (0.09)	-0.08	0.343	-0.04 (0.08)	-0.04	0.660
Education (ref. secondary education)									
Compulsory school	0.22 (0.11)	0.22	0.055	0.20 (0.12)	0.20	0.086	0.15 (0.11)	0.15	0.171
University degree	-0.30 (0.10)	-0.30	0.004	-0.29 (0.10)	-0.29	0.004	-0.25 (0.10)	-0.25	0.010
Proximity to science	-0.14 (0.04)	-0.32	< 0.001	-0.12 (0.04)	-0.30	0.001	-0.10 (0.04)	-0.23	0.008
Urbanity of residence	-0.10 (0.03)	-0.31	< 0.001	-0.10 (0.03)	-0.30	< 0.001	-0.11 (0.03)	-0.32	< 0.001
Swiss region (ref. French-speaking)									
German-speaking	0.08 (0.09)	0.08	0.400	0.09 (0.10)	0.09	0.356	0.11 (0.10)	0.11	0.242
Italian-speaking	0.13 (0.11)	0.13	0.217	0.15 (0.12)	0.15	0.203	0.12 (0.12)	0.12	0.289
Political orientation (1 = left, 7 = right)				0.06 (0.03)	0.16	0.072	0.06 (0.03)	0.16	0.046
Religiosity				0.03 (0.04)	0.07	0.478	0.01 (0.04)	0.03	0.729
Interest in science and research							0.04 (0.04)	0.09	0.333
Scientific literacy							-0.05 (0.01)	-0.36	< 0.001
Trust in science							0.13 (0.09)	0.20	0.122
Trust in scientists							-0.10 (0.06)	-0.16	0.108
Adj. R^2		0.17			0.18			0.21	
F change (df)		—			1.92 (2, 907)	0.146		5.91 (4, 903)	< 0.001
AIC		2712.20			2706.81			2670.44	

Note: *N* = 918. Regressions were run with survey weights using the R package survey v4.1-1. Standardization of *b* estimates follows Gelman's [17] suggestion to rescale the estimates by dividing them by two standard deviations instead of one. Assumption checks, which can be reproduced with the R syntax, neither suggest multicollinearity of explanatory variables nor non-normality or heteroskedasticity of the residuals of any of the regression models.

Table A6. Stepwise multiple linear regressions (DV: conceptions of the academic elite)

	Step 1: Sociodemographic variables			Step 2: Political orientation and religiosity			Step 3: General perceptions of science		
Explanatory Variable	*b (SE)*	β	*p*	*b (SE)*	β	*p*	*b (SE)*	β	*p*
Intercept	3.12 (0.32)	2.76	< 0.001	2.80 (0.31)	2.76	< 0.001	4.21 (0.37)	2.76	< 0.001
Age	0.01 (0.00)	0.26	0.004	0.01 (0.00)	0.20	0.032	0.00 (0.00)	0.12	0.204
Gender (0 = male, 1 = female)	-0.02 (0.09)	-0.02	0.844	-0.03 (0.08)	-0.03	0.728	-0.06 (0.08)	-0.06	0.454
Education (ref. secondary education)									
Compulsory school	-0.23 (0.13)	-0.23	0.079	-0.23 (0.12)	-0.23	0.063	-0.15 (0.12)	-0.15	0.183
University degree	-0.11 (0.09)	-0.11	0.223	-0.09 (0.09)	-0.09	0.304	-0.02 (0.08)	-0.02	0.817
Proximity to science	-0.11 (0.04)	-0.27	0.004	-0.11 (0.04)	-0.26	0.003	-0.09 (0.04)	-0.21	0.019
Urbanity of residence	-0.02 (0.03)	-0.07	0.412	-0.01 (0.03)	-0.04	0.571	-0.01 (0.02)	-0.04	0.585
Swiss region (ref. French-speaking)									
German-speaking	-0.27 (0.11)	-0.27	0.016	-0.30 (0.11)	-0.30	0.004	-0.26 (0.09)	-0.26	0.007
Italian-speaking	-0.49 (0.14)	-0.49	< 0.001	-0.54 (0.13)	-0.54	< 0.001	-0.39 (0.13)	-0.39	0.002
Political orientation (1 = left, 7 = right)				0.03 (0.04)	0.08	0.457	0.03 (0.04)	0.08	0.427
Religiosity				0.10 (0.03)	0.25	0.003	0.06 (0.03)	0.15	0.067
Interest in science and research							0.03 (0.04)	0.06	0.484
Scientific literacy							-0.02 (0.01)	-0.10	0.197
Trust in science							-0.12 (0.08)	-0.18	0.152
Trust in scientists							-0.25 (0.07)	-0.39	< 0.001
Adj. R^2		0.08			0.10			0.18	
F change (df)		—			6.04 (2, 914)	0.002		11.65 (4, 910)	< 0.001
AIC		2690.90			2671.56			2588.77	

Note: *N* = 925. Regressions were run with survey weights using the R package survey v4.1-1. Standardization of *b* estimates follows Gelman's [17] suggestion to rescale the estimates by dividing them by two standard deviations instead of one. Assumption checks, which can be reproduced with the R syntax, neither suggest multicollinearity of explanatory variables nor non-normality or heteroskedasticity of the residuals of any of the regression models.

Table A7. Stepwise multiple linear regressions (DV: demands for decision-making sovereignty)

	Step 1: Sociodemographic variables			Step 2: Political orientation and religiosity			Step 3: General perceptions of science		
Explanatory Variable	*b (SE)*	β	*p*	*b (SE)*	β	*p*	*b (SE)*	β	*p*
Intercept	3.88 (0.39)	2.84	< 0.001	3.28 (0.39)	2.84	< 0.001	2.90 (0.41)	2.84	< 0.001
Age	-0.01 (0.00)	-0.27	0.013	-0.01 (0.00)	-0.34	0.002	-0.01 (0.00)	-0.37	< 0.001
Gender (0 = male, 1 = female)	-0.01 (0.11)	-0.01	0.902	-0.02 (0.10)	-0.02	0.866	0.05 (0.09)	0.05	0.571
Education (ref. secondary education)									
Compulsory school	-0.23 (0.19)	-0.23	0.215	-0.25 (0.17)	-0.25	0.146	-0.31 (0.16)	-0.31	0.059
University degree	-0.20 (0.10)	-0.20	0.052	-0.18 (0.10)	-0.18	0.076	-0.19 (0.10)	-0.19	0.064
Proximity to science	-0.10 (0.05)	-0.24	0.030	-0.08 (0.04)	-0.20	0.063	-0.09 (0.04)	-0.21	0.044
Urbanity of residence	-0.04 (0.03)	-0.11	0.215	-0.03 (0.03)	-0.08	0.332	-0.04 (0.03)	-0.13	0.111
Swiss region (ref. French-speaking)									
German-speaking	-0.09 (0.14)	-0.09	0.505	-0.08 (0.12)	-0.08	0.498	-0.03 (0.12)	-0.03	0.793
Italian-speaking	-0.13 (0.19)	-0.13	0.495	-0.12 (0.18)	-0.12	0.494	-0.17 (0.18)	-0.17	0.344
Political orientation (1 = left, 7 = right)				0.10 (0.04)	0.28	0.012	0.11 (0.04)	0.28	0.007
Religiosity				0.08 (0.04)	0.19	0.083	0.08 (0.04)	0.19	0.058
Interest in science and research							0.17 (0.04)	0.35	< 0.001
Scientific literacy							-0.04 (0.01)	-0.24	0.007
Trust in science							0.06 (0.08)	0.09	0.421
Trust in scientists							-0.04 (0.08)	-0.06	0.622
Adj. R^2		0.05			0.07			0.11	
F change (df)		—			5.20 (2, 914)	0.006		5.05 (4, 910)	< 0.001
AIC		2954.34			2929.17			2895.30	

Note: *N* = 925. Regressions were run with survey weights using the R package survey v4.1-1. Standardization of *b* estimates follows Gelman's [17] suggestion to rescale the estimates by dividing them by two standard deviations instead of one. Assumption checks, which can be reproduced with the R syntax, neither suggest multicollinearity of explanatory variables nor non-normality or heteroskedasticity of the residuals of any of the regression models.

Table A8. Stepwise multiple linear regressions (DV: demands for truth-speaking sovereignty)

	Step 1: Sociodemographic variables			Step 2: Political orientation and religiosity			Step 3: General perceptions of science		
Explanatory Variable	*b (SE)*	β	*p*	*b (SE)*	β	*p*	*b (SE)*	β	*p*
Intercept	3.32 (0.32)	3.14	< 0.001	2.95 (0.35)	3.14	< 0.001	4.59 (0.42)	3.14	< 0.001
Age	0.02 (0.00)	0.57	< 0.001	0.01 (0.00)	0.51	< 0.001	0.01 (0.00)	0.42	< 0.001
Gender (0 = male, 1 = female)	0.08 (0.09)	0.08	0.400	0.07 (0.09)	0.07	0.452	0.03 (0.09)	0.03	0.711
Education (ref. secondary education)									
Compulsory school	-0.21 (0.15)	-0.21	0.153	-0.21 (0.14)	-0.21	0.129	-0.15 (0.13)	-0.15	0.272
University degree	-0.39 (0.10)	-0.39	< 0.001	-0.37 (0.10)	-0.37	< 0.001	-0.28 (0.09)	-0.28	0.003
Proximity to science	-0.18 (0.04)	-0.43	< 0.001	-0.18 (0.04)	-0.42	< 0.001	-0.13 (0.04)	-0.31	0.002
Urbanity of residence	-0.05 (0.03)	-0.16	0.072	-0.05 (0.03)	-0.14	0.125	-0.04 (0.03)	-0.13	0.156
Swiss region (ref. French-speaking)									
German-speaking	0.04 (0.12)	0.04	0.722	0.02 (0.11)	0.02	0.844	0.06 (0.10)	0.06	0.563
Italian-speaking	-0.34 (0.15)	-0.34	0.024	-0.37 (0.14)	-0.37	0.009	-0.21 (0.12)	-0.21	0.089
Political orientation (1 = left, 7 = right)				0.04 (0.04)	0.12	0.309	0.04 (0.04)	0.12	0.305
Religiosity				0.09 (0.04)	0.22	0.026	0.04 (0.03)	0.10	0.255
Interest in science and research							-0.03 (0.04)	-0.07	0.425
Scientific literacy							-0.03 (0.01)	-0.22	0.012
Trust in science							-0.13 (0.08)	-0.19	0.109
Trust in scientists							-0.23 (0.07)	-0.35	0.002
Adj. R²		0.21			0.22			0.30	
F change (df)		—			4.41 (2, 912)	0.012		13.62 (4, 908)	< 0.001
AIC		2781.42			2766.44			2676.75	

Note: N = 923. Regressions were run with survey weights using the R package survey v4.1-1. Standardization of *b* estimates follows Gelman's [17] suggestion to rescale the estimates by dividing them by two standard deviations instead of one. Assumption checks, which can be reproduced with the R syntax, neither suggest multicollinearity of explanatory variables nor non-normality or heteroskedasticity of the residuals of any of the regression models.

Table A9. Quadratic multiple linear regressions (DV: science-related populist attitudes)

Explanatory Variable	*b (SE)*	β	*p*
Intercept	3.26 (0.44)	2.21	< 0.001
Age	0.00 (0.00)	0.01	0.945
Gender (0 = male, 1 = female)	0.07 (0.08)	0.07	0.397
Education (ref. secondary education)			
Compulsory school	-0.38 (0.14)	-0.38	0.007
University degree	-0.17 (0.08)	-0.17	0.030
Proximity to science	-0.1 (0.03)	-0.25	0.002
Urbanity of residence	-0.02 (0.02)	-0.05	0.501
Swiss region (ref. French-speaking)			
German-speaking	-0.16 (0.10)	-0.16	0.122
Italian-speaking	-0.23 (0.11)	-0.23	0.038
Political orientation	-0.11 (0.16)	-0.30	0.483
Political orientation × political orientation	0.03 (0.02)	0.55	0.266
Religiosity	0.03 (0.03)	0.06	0.455
Interest in science and research	0.08 (0.03)	0.17	0.011
Scientific literacy	-0.04 (0.01)	-0.28	< 0.001
Trust in science	-0.05 (0.08)	-0.07	0.532
Trust in scientists	-0.13 (0.07)	-0.20	0.064
Adj. R^2		0.18	
F (df)		9.96 (15, 899)	< 0.001
AIC		2428.35	

Note: *N* = 915. Regressions were run with survey weights using the R package survey v4.1-1. Standardization of *b* estimates follows Gelman's [17] suggestion to rescale the estimates by dividing them by two standard deviations instead of one. Assumption checks, which can be reproduced with the R syntax, did not suggest non-normality or heteroskedasticity of the residuals.

Table A10. Multiple linear regressions explaining science-related populist attitudes (sensitivity test 1: Bollenian instead of Goertzian SciPop Scores).

Explanatory Variable	*b (SE)*	β	*p*
Intercept	3.87 (0.28)	3.01	< 0.001
Age	0.00 (0.00)	0.11	0.090
Gender (0 = male, 1 = female)	0.00 (0.06)	0.00	0.979
Education (ref. secondary education)			
Compulsory school	-0.11 (0.09)	-0.11	0.223
University degree	-0.18 (0.06)	-0.18	0.002
Proximity to science	-0.10 (0.03)	-0.23	< 0.001
Urbanity of residence	-0.05 (0.02)	-0.16	0.005
Swiss region (ref. French-speaking)			
German-speaking	-0.03 (0.07)	-0.03	0.665
Italian-speaking	-0.15 (0.09)	-0.15	0.108
Political orientation	0.06 (0.02)	0.16	0.011
Religiosity	0.05 (0.02)	0.12	0.041
Interest in science and research	0.05 (0.03)	0.11	0.052
Scientific literacy	-0.04 (0.01)	-0.23	< 0.001
Trust in science	-0.01 (0.05)	-0.02	0.826
Trust in scientists	-0.16 (0.05)	-0.24	0.002
Adj. R^2		0.26	
F (df)		16.19 (14, 911)	< 0.001
AIC		1977.57	

Note: N = 915. Regressions were run with survey weights using the R package survey v4.1-1. Standardization of *b* estimates follows Gelman's [17] suggestion to rescale the estimates by dividing them by two standard deviations instead of one.

Table A11. Multiple linear regressions explaining science-related populist attitudes and its components (sensitivity test 2: trimmed instead of original weights).

	Science-related populist attitudes			*Conceptions of the ordinary people*			*Conceptions of the academic elite*			*Demands for decision-making sovereignty*			*Demands for truth-speaking sovereignty*		
Explanatory Variable	*b (SE)*	*β*	*p*	*b (SE)*	*β*	*p*	*b (SE)*	*β*	*p*	*b (SE)*	*β*	*p*	*b (SE)*	*β*	*p*
Intercept	3.07 (0.38)	2.18	< 0.001	3.60 (0.35)	3.27	< 0.001	4.33 (0.35)	2.75	< 0.001	3.03 (0.40)	2.83	< 0.001	4.68 (0.38)	3.11	< 0.001
Age	-0.00 (0.00)	-0.01	0.873	0.01 (0.00)	0.29	0.001	0.00 (0.00)	0.12	0.193	-0.01 (0.00)	-0.35	< 0.001	0.01 (0.00)	0.38	< 0.001
Gender (0 = male, 1 = female)	0.00 (0.07)	0.00	0.973	-0.05 (0.08)	-0.05	0.543	-0.10 (0.07)	-0.10	0.188	0.03 (0.09)	0.03	0.768	0.00 (0.08)	0.00	0.961
Education (ref. secondary education)															
Compulsory school	-0.43 (0.14)	-0.43	0.002	0.11 (0.11)	0.11	0.339	-0.21 (0.12)	-0.21	0.071	-0.27 (0.16)	-0.27	0.088	-0.20 (0.13)	-0.20	0.121
University degree	-0.19 (0.07)	-0.19	0.010	-0.28 (0.09)	-0.28	0.001	-0.03 (0.08)	-0.03	0.728	-0.22 (0.09)	-0.22	0.014	-0.30 (0.08)	-0.30	< 0.001
Proximity to science	-0.09 (0.03)	-0.22	0.003	-0.10 (0.03)	-0.25	0.002	-0.08 (0.03)	-0.18	0.025	-0.07 (0.04)	-0.18	0.065	-0.12 (0.04)	-0.29	0.001
Urbanity of residence	-0.03 (0.02)	-0.08	0.278	-0.09 (0.02)	-0.29	< 0.001	-0.03 (0.02)	-0.08	0.263	-0.04 (0.03)	-0.14	0.078	-0.05 (0.03)	-0.15	0.059
Swiss region (ref. French-speaking)															
German-speaking	-0.06 (0.09)	-0.06	0.511	0.13 (0.09)	0.13	0.165	-0.17 (0.09)	-0.17	0.050	-0.04 (0.11)	-0.04	0.741	0.11 (0.09)	0.11	0.217
Italian-speaking	-0.14 (0.10)	-0.14	0.170	0.14 (0.11)	0.14	0.208	-0.30 (0.12)	-0.30	0.008	-0.18 (0.16)	-0.18	0.254	-0.13 (0.11)	-0.13	0.217
Political orientation (1 = left, 7 = right)	0.05 (0.03)	0.14	0.089	0.06 (0.03)	0.16	0.039	0.01 (0.03)	0.03	0.738	0.08 (0.04)	0.21	0.022	0.02 (0.03)	0.05	0.539
Religiosity	0.03 (0.03)	0.08	0.310	0.01 (0.03)	0.02	0.777	0.07 (0.03)	0.17	0.018	0.08 (0.04)	0.19	0.037	0.05 (0.03)	0.13	0.082
Interest in science and research	0.07 (0.03)	0.15	0.027	0.04 (0.04)	0.09	0.294	0.03 (0.04)	0.05	0.480	0.16 (0.04)	0.35	< 0.001	-0.05 (0.04)	-0.10	0.224
Scientific literacy	-0.04 (0.01)	-0.28	< 0.001	-0.05 (0.01)	-0.34	< 0.001	-0.02 (0.01)	-0.12	0.106	-0.04 (0.01)	-0.24	0.004	-0.03 (0.01)	-0.23	0.006
Trust in science	0.00 (0.07)	0.00	0.972	0.16 (0.07)	0.24	0.022	-0.10 (0.07)	-0.15	0.144	0.04 (0.07)	0.06	0.591	-0.08 (0.07)	-0.12	0.252
Trust in scientists	-0.16 (0.06)	-0.25	0.014	-0.11 (0.06)	-0.17	0.074	-0.26 (0.06)	-0.41	< 0.001	-0.03 (0.07)	-0.05	0.654	-0.25 (0.07)	-0.40	< 0.001
Adj. R²		0.15			0.20			0.17			0.10			0.28	
F (df)		10.62 (14, 900)	< 0.001		11.50 (14, 903)	< 0.001		8.04 (14, 910)	< 0.001		4.77 (14, 910)	< 0.001		18.74 (14, 908)	< 0.001
AIC		2304.33			2568.53			2499.35			2800.40			2564.99	
N		915			918			925			925			923	

Note: Values indicated are standardized regression coefficients. Regressions were run with survey weights using the R package survey v4.1-1 [1]. Standardization of *b* coefficients follows Gelman's [17] suggestion to rescale the estimates by dividing them by two standard deviations instead of one.

Supplementary Figures

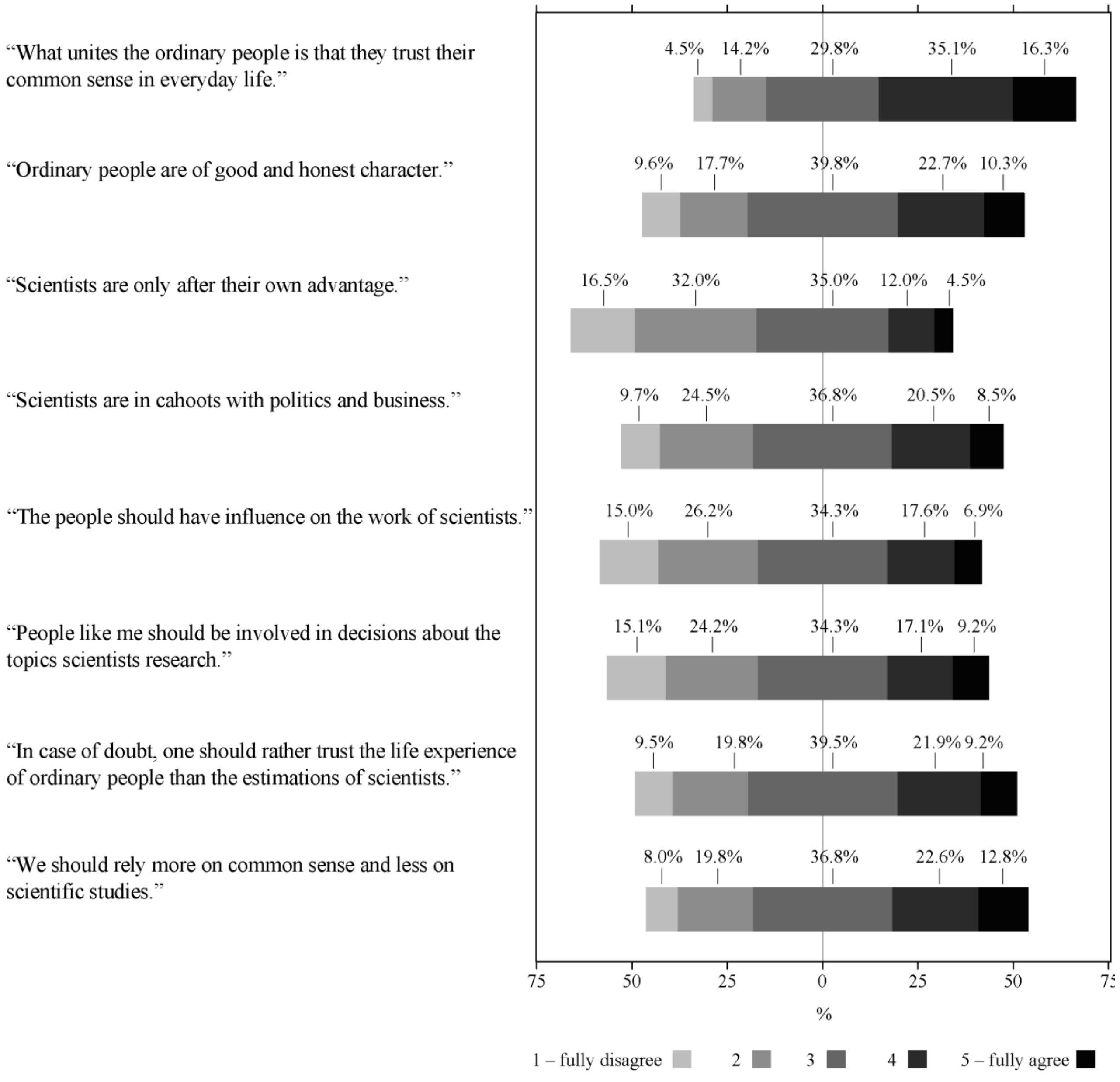

Fig A1. Relative response frequencies of SciPop Scale items

Note. Analyses based on weighted data.

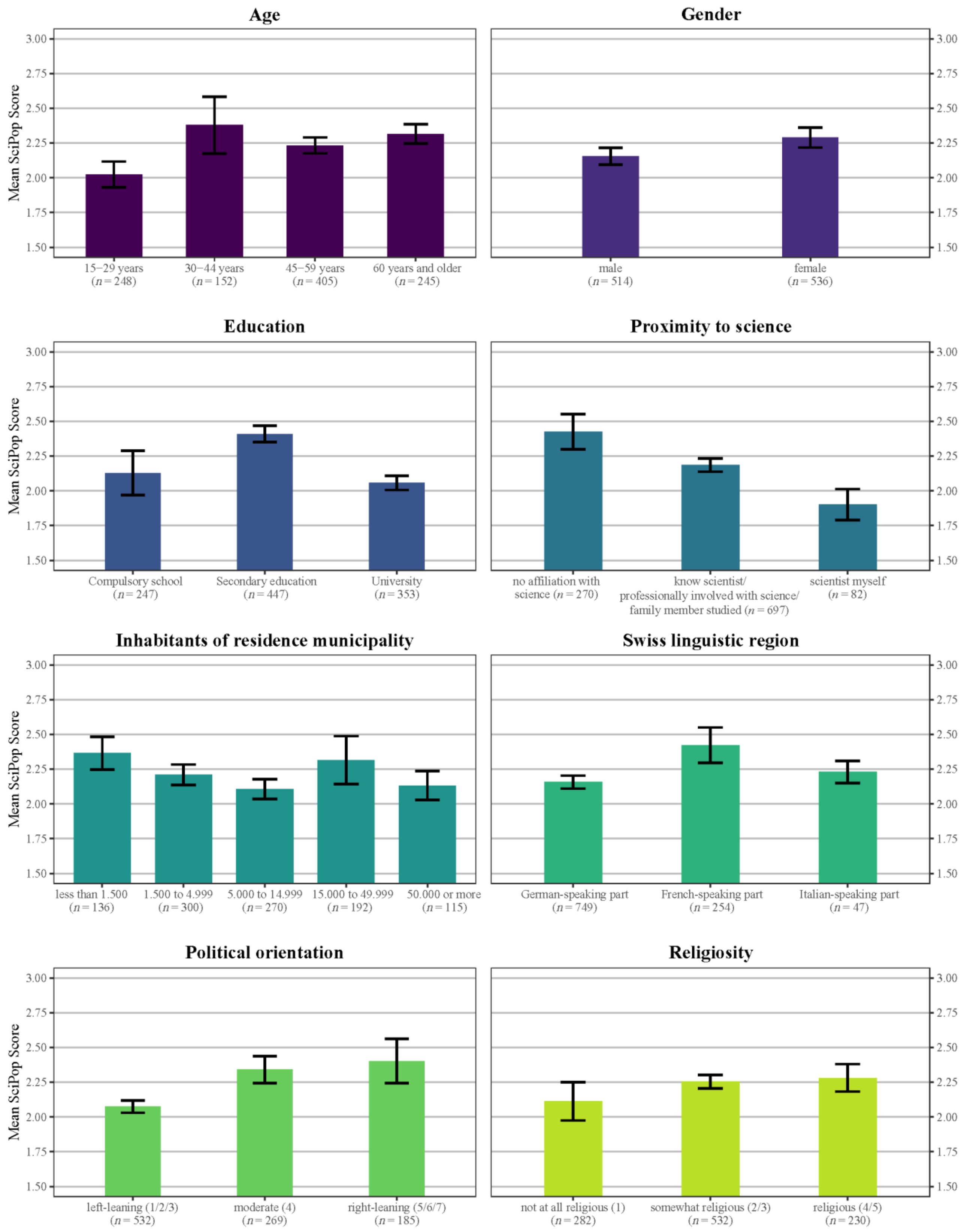

Fig A2. Mean SciPop Scores in sample subgroups

Note. Error bars represent standard errors. Analyses based on weighted data.

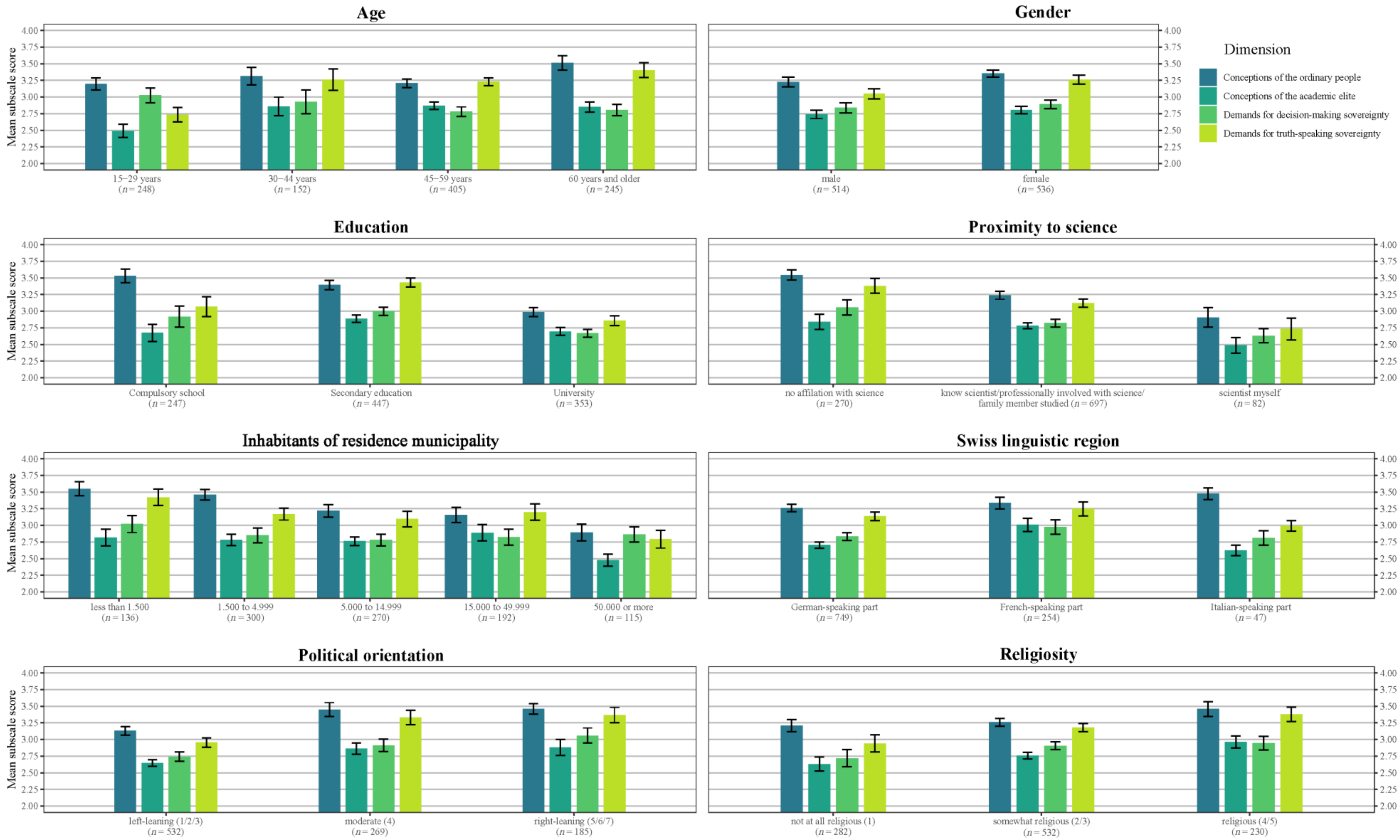

Fig A3. Means of subscale scores in sample subgroups

Note. Error bars represent standard errors. Analyses based on weighted data.

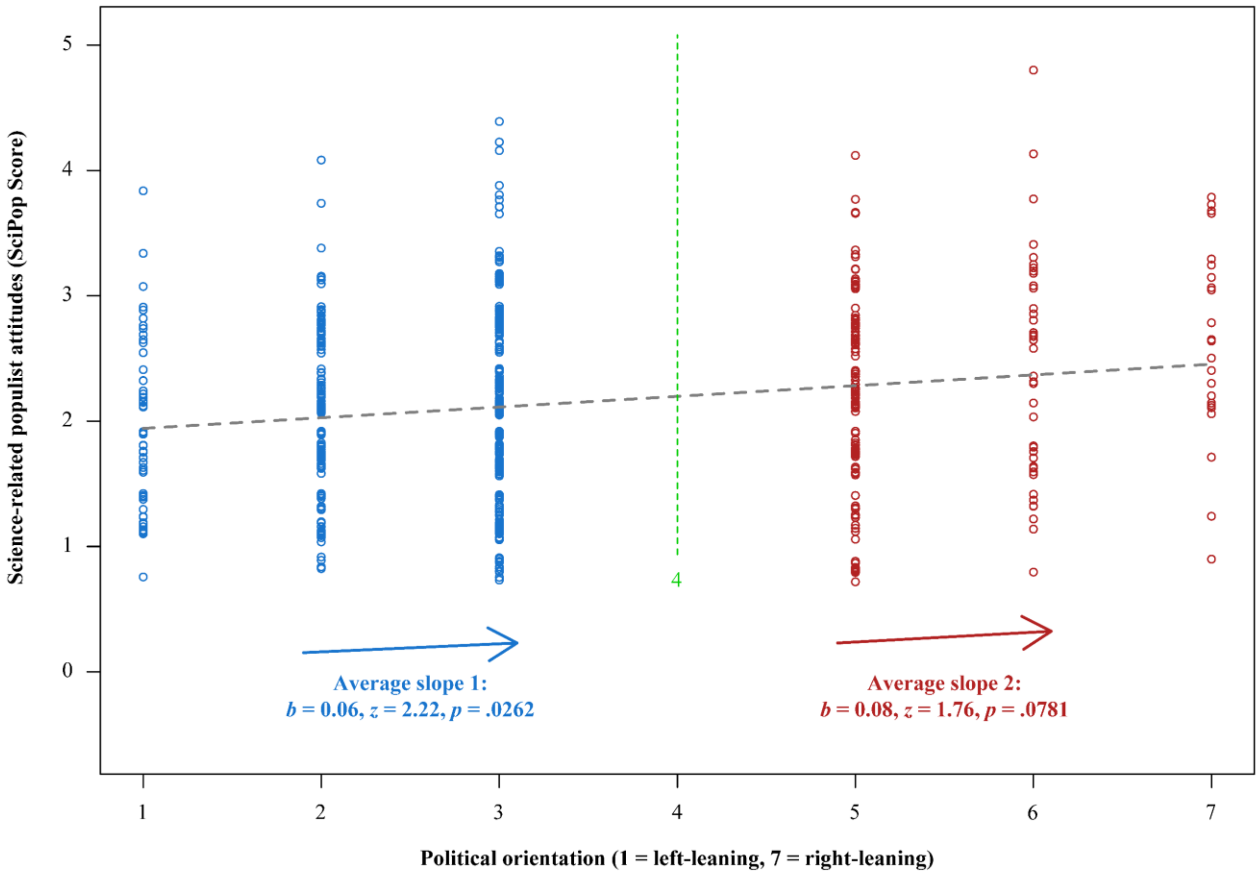

Fig A4. Plot of two-lines test of relationship between SciPop Score and political orientation

Note. Analysis was not based on weighted data. Covariates: Age, gender, education, proximity to science, urbanity of residence, Swiss region, religiosity, interest in science and research, scientific literacy, trust in science, trust in scientists.

References

1. Lumley T. Package ‘survey’. Analysis of complex survey samples. R package version 4.1-1. 2021. Available from: https://cran.r-project.org/web/packages/survey/.

2. Simonsohn U. Two lines: A valid alternative to the invalid testing of U-shaped relationships with quadratic regressions. Adv Methods Pract Psychol Sci. 2018; 1:538–55. doi: 10.1177/2515245918805755.

3. Field A, Miles J, Field Z. Discovering statistics using R. Los Angeles: Sage; 2012.

4. Menard S. Applied logistic regression analysis. Thousand Oaks: Sage; 1995.

5. Wuttke A, Schimpf C, Schoen H. When the whole is greater than the sum of its parts. On the conceptualization and measurement of populist attitudes and other multidimensional constructs. Am Polit Sci Rev. 2020; 114:356–74. doi: 10.1017/S0003055419000807.

6. Mede NG, Schäfer MS, Füchslin T. The SciPop Scale for measuring science-related populist attitudes in surveys. Development, test, and validation. Int J Public Opin Res. 2021; 33:273–93. doi: 10.1093/ijpor/edaa026.

7. Stier S, Kirkizh N, Froio C, Schroeder R. Populist attitudes and selective exposure to online news. A cross-country analysis combining web tracking and surveys. Int J Press Polit. 2020; 25:426-446. doi: 10.1177/1940161220907018.

8. Dekeyser D, Roose H. Unpacking populism: Using correlational class analysis to understand how people interrelate populist, pluralist, and elitist attitudes. Swiss Polit Sci Rev. 2021; 27:476–95. doi: 10.1111/spsr.12463.

9. Erisen C, Guidi M, Martini S, Toprakkiran S, Isernia P, Littvay L. Psychological correlates of populist attitudes. Polit Psychol. 2021; 42:149–71. doi: 10.1111/pops.12768.

10. Leeuw ED de, Hox J, Dillman D, editors. International handbook of survey methodology. 1st ed. New York: Routledge; 2008.

11. Biemer PP, Christ SL. Weighting survey data. In: Leeuw ED de, Hox J, Dillman D, editors. International handbook of survey methodology. New York: Routledge; 2008. pp. 317–41.

12. Liu B, Ferraro D, Wilson E, Brick JM. Trimming extreme survey weights in household surveys. Proceedings of the Section on Survey Research Methods. American Statistical Association, Alexandria, VA 2004.

13. van de Kerckhove W, Mohadjer L, Krenzke T. A weight-trimming approach to achieve a comparable increase to bias across countries in the Programme for the International Assessment of Adult Competencies. Proceedings of the Section on Survey Research Methods. American Statistical Association, Alexandria, VA 2014.

14. Chowdhury S, Khare M, Wolter K. Weight trimming in the National Immunization Survey. Proceedings of the Section on Survey Research Methods. American Statistical Association, Alexandria, VA 2007.

15. Potter F, Zheng Y. Methods and issues in trimming extreme weights in sample surveys. Proceedings of the Section on Survey Research Methods. American Statistical Association, Alexandria, VA 2015.

16. Decker EH, Kerkhoff AJ, Moses ME. Global patterns of city size distributions and their fundamental drivers. PLoS One. 2007; 2. doi: 10.1371/journal.pone.0000934.

17. Gelman A. Scaling regression inputs by dividing by two standard deviations. Stat Med. 2008; 27:2865–73. doi: 10.1002/sim.3107.
