# Supplementary material for: Who supports science-related populism? A nationally representative survey on the prevalence and explanatory factors of populist attitudes toward science in Switzerland
Source: PLoS One. 2022 Aug 8;17(8):e0271204. doi: 10.1371/journal.pone.0271204 (PMC9359586; doi:10.1371/journal.pone.0271204)
Supplement: S2 Appendix. Original questionnaires in German, French, and Italian — (PDF) [file pone.0271204.s002.pdf]

Einstieg

1. Alter[1]

In welchem Jahr sind Sie geboren? ___________

2. Geschlecht

☐ 1 Männlich
☐ 2 Weiblich

3. Wohnort

Wie lautet die Postleitzahl Ihres Wohnortes? ___________

4. Interesse an aktuellen Themen

Ich lese Ihnen jetzt verschiedene Themen vor. Bitte sagen Sie mir auf einer Skala von 1 bis 5, wie stark Sie sich für jedes Thema interessieren. 1 bedeutet «überhaupt nicht» und 5 bedeutet «sehr stark».

	1 überhaupt nicht	2	3	4	5 Sehr stark
Politik im In- und Ausland	☐	☐	☐	☐	☐
Wirtschaft und Finanzen	☐	☐	☐	☐	☐
Sport	☐	☐	☐	☐	☐
Wissenschaft und Forschung	☐	☐	☐	☐	☐
Menschliche Schicksale, Unglücksfälle, Verbrechen, etc.	☐	☐	☐	☐	☐

[1] „Weiss nicht“ (98) und „keine Angabe“ (99) war bei allen Fragen eine Antwortoption.

5. Forschungsthemen

Wir möchten Sie im Folgenden weiter zu Ihrer Meinung zu Wissenschaft und Forschung befragen. Was meinen Sie: Wie stark sollte die Wissenschaft in den nächsten 15 Jahren die folgenden Themen erforschen? Sie können wieder auf einer Skala von 1 bis 5 antworten. 1 bedeutet «überhaupt nicht» und 5 bedeutet «sehr stark».

	1<br>Überhaupt nicht	2	3	4	5<br>Sehr stark
Klima und Energie	☐	☐	☐	☐	☐
Gesundheit und Ernährung	☐	☐	☐	☐	☐
Verkehr und Mobilität	☐	☐	☐	☐	☐
Kommunikation und Digitalisierung	☐	☐	☐	☐	☐
Wirtschaftliche Entwicklung und Arbeitsmarkt	☐	☐	☐	☐	☐
Einwanderung und Integration	☐	☐	☐	☐	☐

Mediennutzung und Informationsverhalten

6. Häufigkeit der Informationsnutzung

Wie häufig kommen Sie durch Medien mit Wissenschaft und Forschung in Kontakt?
Sie können wieder auf einer Skala von 1 bis 5 antworten. 1 bedeutet «nie», 5 bedeutet «sehr häufig».

6.1. Offline Informationsnutzung

	1 nie	2	3	4	5 Sehr häufig
Fernsehen, ohne Online-Mediatheken	☐	☐	☐	☐	☐
und davon beim Schweizer Fernsehen, SRF	☐	☐	☐	☐	☐
Radio	☐	☐	☐	☐	☐
und davon beim Schweizer Radio, SRF	☐	☐	☐	☐	☐
Gedruckte Tageszeitungen, Wochenzeitungen oder Zeitschriften	☐	☐	☐	☐	☐
Wissenschaftsmagazine wie «PM» oder «Spektrum der Wissenschaft»	☐	☐	☐	☐	☐
im Internet	☐	☐	☐	☐	☐

6.2. Online Informationsnutzung

FILTER: WENN Internet genutzt=2-5

	1 nie	2	3	4	5 Sehr häufig
Webseiten oder Apps von Zeitungen und Zeitschriften	☐	☐	☐	☐	☐
Mediatheken von Fernseh- und Radiosendern	☐	☐	☐	☐	☐
Webseiten von wissenschaftlichen Einrichtungen, Behörden und Organisationen	☐	☐	☐	☐	☐
Facebook, Twitter oder andere soziale Netzwerke	☐	☐	☐	☐	☐
Blogs oder Online-Foren	☐	☐	☐	☐	☐
Wikipedia	☐	☐	☐	☐	☐
YouTube oder ähnliche Videoplattformen	☐	☐	☐	☐	☐

7. Konative Einstellungen: Social Media-Kommunikation

FILTER: WENN Internet genutzt=2-5

Bitte sagen Sie mir, wie häufig Sie Folgendes im Internet machen. 1 bedeutet «nie», 5 bedeutet «sehr häufig».

	1<br>nie	2	3	4	5<br>sehr häufig
Informationen oder Meinungen über Wissenschaft und Forschung posten oder teilen	☐	☐	☐	☐	☐
Informationen oder Meinungen über Wissenschaft und Forschung liken oder favorisieren	☐	☐	☐	☐	☐
Informationen oder Meinungen über Wissenschaft und Forschung kommentieren	☐	☐	☐	☐	☐

8. Aufmerksamkeit für Wissenschaft in Medien

Wir haben noch ein paar weitere Fragen zur Berichterstattung über Wissenschaft und Forschung in den Medien. Wie aufmerksam verfolgen Sie diese? 1 bedeutet «überhaupt nicht aufmerksam», 5 bedeutet «sehr aufmerksam».

1<br>Überhaupt nicht aufmerksam	2	3	4	5<br>Sehr aufmerksam
☐	☐	☐	☐	☐

9. Zufriedenheit mit Medienberichterstattung

Und ganz generell, wie zufrieden sind Sie mit der Medienberichterstattung über Wissenschaft und Forschung? 1 bedeutet «überhaupt nicht zufrieden», 5 bedeutet «sehr zufrieden».

1<br>Überhaupt nicht zufrieden	2	3	4	5<br>Sehr zufrieden
☐	☐	☐	☐	☐

10. Bewertung der Medienberichterstattung

Wie stark stimmen Sie folgenden Aussagen zu? Die Medienberichterstattung über Wissenschaft und Forschung ist im Allgemeinen…

	1<br>Stimme überhaupt nicht zu	2	3	4	5<br>Stimme voll und ganz zu
vertrauenswürdig	☐	☐	☐	☐	☐
verständlich	☐	☐	☐	☐	☐
ausführlich	☐	☐	☐	☐	☐
Stellt Wissenschaft und Forschung negativ dar	☐	☐	☐	☐	☐

11. Nutzung nicht-massenmedialer Informationsquellen

Mit Wissenschaft und Forschung kann man nicht nur in Medien, sondern auch auf andere Weise in Kontakt kommen. Ich nenne Ihnen einige Aktivitäten. Bitte sagen Sie mir, wie häufig Sie das machen. 1 bedeutet «nie», 5 bedeutet «sehr häufig».

	1<br>nie	2	3	4	5<br>Sehr häufig
Museen und Ausstellungen zu Wissenschaft und Forschung besuchen	☐	☐	☐	☐	☐
Zoos, Aquarien oder Botanische Gärten besuchen	☐	☐	☐	☐	☐
Veranstaltungen, Vorträge und Diskussionen zu Wissenschaft und Forschung besuchen	☐	☐	☐	☐	☐
Filme und Serien anschauen, in denen es auch um Wissenschaft und Forschung geht	☐	☐	☐	☐	☐
mit Freunden und Bekannten über Wissenschaft und Forschung sprechen	☐	☐	☐	☐	☐
Mich in Messengern wie WhatsApp über Wissenschaft und Forschung austauschen	☐	☐	☐	☐	☐

12. **Heuristische und systematische Informationsverarbeitung und perceived behavioral control**

Jetzt würden wir gerne Ihre Meinung über Wissenschaft und Forschung hören. 1 bedeutet «stimme überhaupt nicht zu» und 5 bedeutet «stimme voll und ganz zu».

	1<br>Stimme überhaupt nicht zu	2	3	4	5<br>Stimme voll und ganz zu
Ich bin gut über Wissenschaft und Forschung informiert	☐	☐	☐	☐	☐
Ich suche gezielt Informationen über Wissenschaft und Forschung	☐	☐	☐	☐	☐
Ich weiss viel über Wissenschaft und Forschung.	☐	☐	☐	☐	☐
Es ist wichtig, dass man über Wissenschaft und Forschung informiert ist.	☐	☐	☐	☐	☐

Wahrnehmung von Wissenschaft

13. Ziele von Wissenschaft

Über die Funktion von Wissenschaft gibt es unterschiedliche Meinungen. Ich lese Ihnen einige Aussagen vor. Bitte sagen Sie mir, wie stark Sie diesen zustimmen.

	1 Stimme überhaupt nicht zu	2	3	4	5 Stimme voll und ganz zu
Wissenschaftliche Forschung ist notwendig, auch wenn sich daraus kein unmittelbarer Nutzen ergibt.	☐	☐	☐	☐	☐
Wissenschaftliche Forschung sollte staatlich unterstützt werden.	☐	☐	☐	☐	☐
Wissenschaftler sollten die Öffentlichkeit über ihre Arbeit informieren.	☐	☐	☐	☐	☐
Wissenschaftler sollten mehr darauf hören, was einfache Leute denken.	☐	☐	☐	☐	☐
Politische Entscheidungen sollten auf wissenschaftlichen Erkenntnissen beruhen.	☐	☐	☐	☐	☐
Leute wie ich sollten mitentscheiden, zu welchen Themen Wissenschaftler forschen.	☐	☐	☐	☐	☐
Ich würde gern einmal in wissenschaftlichen Projekten mitforschen.	☐	☐	☐	☐	☐
Wissenschaft und Forschung spielen in meinem Leben eine wichtige Rolle.	☐	☐	☐	☐	☐

14. Reservations vs. beliefs in the promise of science

Was glauben Sie: Welche Auswirkungen haben Wissenschaft und Forschung auf unser Leben?

	1 Stimme überhaupt nicht zu	2	3	4	5 Stimme voll und ganz zu
Wissenschaft und Forschung können jedes Problem lösen.	☐	☐	☐	☐	☐
Wissenschaft und Forschung verbessern unser Leben.	☐	☐	☐	☐	☐
Durch Wissenschaft und Forschung ändert sich unser Leben zu schnell.	☐	☐	☐	☐	☐
Der Nutzen von Wissenschaft und Forschung ist grösser als die möglicherweise auftretenden Schäden.	☐	☐	☐	☐	☐
Die Wissenschaft sollte ohne Einschränkung alles erforschen dürfen.	☐	☐	☐	☐	☐
Die Wissenschaft wird uns eines Tages ein vollständiges Bild davon vermitteln, wie Natur und Universum funktionieren.	☐	☐	☐	☐	☐
Wir verlassen uns zu sehr auf die Wissenschaft.	☐	☐	☐	☐	☐

15. Vertrauen in Wissenschaft

Auf einer Skala von 1 bis 5, wo 1 «sehr gering» und 5 «sehr hoch» bedeutet, was würden Sie sagen, wie hoch ist Ihr Vertrauen …

	1 Sehr gering	2	3	4	5 Sehr hoch
... in Wissenschaftlerinnen und Wissenschaftler an Universitäten?	☐	☐	☐	☐	☐
…in die Wissenschaft allgemein?	☐	☐	☐	☐	☐

16. Vertrauen in WissenschaftlerInnen (METI)

Welche Eigenschaften bringen Sie mit Wissenschaftlerinnen und Wissenschaftlern in Bezug auf Ihre Forschung in Verbindung? Wir nennen Ihnen je eine Eigenschaft und ihr Gegenteil. Wenn sie denken, dass diese Eigenschaft voll auf Wissenschaftler zutrifft, vergeben Sie eine 5. Wenn sie denken, dass diese Eigenschaft ganz und gar nicht auf Wissenschaftler zutrifft, vergeben Sie eine 1. Mit den Zahlen dazwischen können Sie Ihre Meinung abstufen.

	1	2	3	4	5	
inkompetent	☐	☐	☐	☐	☐	kompetent
unerfahren	☐	☐	☐	☐	☐	erfahren
unqualifiziert	☐	☐	☐	☐	☐	qualifiziert
unaufrichtig	☐	☐	☐	☐	☐	aufrichtig
unehrlich	☐	☐	☐	☐	☐	ehrlich
ungerecht	☐	☐	☐	☐	☐	gerecht
unmoralisch	☐	☐	☐	☐	☐	moralisch
unethisch	☐	☐	☐	☐	☐	ethisch
verantwortungslos	☐	☐	☐	☐	☐	verantwortungsbewusst

17. Science Populism Scale

Nun geht es um das Verhältnis von Wissenschaft und Gesellschaft. Wie stark stimmen Sie den folgenden Aussagen zu?

	1 Stimme überhaupt nicht zu	2	3	4	5 Stimme voll und ganz zu
Das Volk sollte Einfluss auf die Arbeit von Wissenschaftlern haben.	☐	☐	☐	☐	☐
Im Zweifel sollte man eher der Lebenserfahrung einfacher Menschen vertrauen als Einschätzungen von Wissenschaftlern.	☐	☐	☐	☐	☐
Wir sollten uns mehr auf den gesunden Menschenverstand und weniger auf wissenschaftliche Studien verlassen.	☐	☐	☐	☐	☐
Wissenschaftler sind nur auf ihren eigenen Vorteil aus.	☐	☐	☐	☐	☐
Wissenschaftler stecken mit Politik und Wirtschaft unter einer Decke.	☐	☐	☐	☐	☐
Die einfachen Leute teilen gemeinsame Werte und Interessen.	☐	☐	☐	☐	☐
Was die einfachen Leute verbindet, ist, dass sie im Alltag ihrem gesunden Menschenverstand trauen.	☐	☐	☐	☐	☐
Einfache Leute verbindet ein guter und ehrlicher Charakter.	☐	☐	☐	☐	☐
Die Wissenschaft ist Teil der Elite unseres Landes.	☐	☐	☐	☐	☐

18. Scientific Literacy

Ich lese Ihnen jetzt einige Aussagen vor, die Sie möglicherweise aus der Schule oder den Medien kennen. Einige sind falsch, einige richtig. Bitte sagen Sie uns, ob diese Aussagen Ihrer Ansicht nach richtig oder falsch sind und ob Sie sich dabei sicher sind. Wenn Sie es bei einer Aussage nicht wissen, sagen Sie mir das ruhig. Wir machen dann mit der nächsten Aussage weiter.

	1 sicher falsch	2 eher falsch	3 eher richtig	4 sicher richtig	98 weiss nicht
Die Kontinente, auf denen wir leben, bewegen sich schon seit Millionen von Jahren. (richtig)	☐	☐	☐	☐	☐
Elektronen sind kleiner als Atome. (richtig)	☐	☐	☐	☐	☐
Antibiotika töten sowohl Viren als auch Bakterien. (falsch)	☐	☐	☐	☐	☐
Die Gene von der Mutter entscheiden, ob ein Kind ein Bube oder ein Mädchen wird. (falsch)	☐	☐	☐	☐	☐
Wissenschaftliche Theorien ändern sich nie. (falsch)	☐	☐	☐	☐	☐

19. Systembezogene Medienverdrossenheit

Unabhängig vom Thema Wissenschaft: Was denken Sie allgemein über die Medien in der Schweiz? Mit Medien sind etablierte Nachrichtenmedien gemeint, die über das aktuelle Geschehen informieren. Bitte geben Sie an, ob Sie den folgenden Aussagen eher zustimmen oder nicht.

	1 Stimme überhaupt nicht zu	2	3	4	5 Stimme voll und ganz zu
Das System der Nachrichtenmedien halte ich für korrupt.	☐	☐	☐	☐	☐
Die Nachrichtenmedien handeln vor allem in eigenem Interesse.	☐	☐	☐	☐	☐
Was die Nachrichtenmedien treiben, macht mich wütend.	☐	☐	☐	☐	☐
Die Nachrichtenmedien helfen dabei die wichtigen Probleme der Gesellschaft zu lösen.	☐	☐	☐	☐	☐

Persönlichkeitsvariablen und Soziodemographie

20. Direkte Erfahrungen mit Wissenschaft

Zum Abschluss des Interviews hätten wir noch einige Fragen zu Ihrer Person.

a. Sind Sie selber WissenschaftlerIn?

- ☐ 1 Ja
- ☐ 2 Nein

Filter: NUR wenn F20 a. = 2

	1<br>Ja	2<br>Nein
Kennen Sie eine Wissenschaftlerin oder einen Wissenschaftler persönlich?	☐	☐
Haben Sie beruflich mit Wissenschaft zu tun?	☐	☐
Haben Sie Familienmitglieder, die studiert haben oder noch studieren?	☐	☐

21. Bildung

Welche Ausbildung haben Sie zuletzt abgeschlossen?

- ☐ 1 **Keine Ausbildung abgeschlossen** (Keine Ausbildung / Primarschule, bis 7 Jahre obligatorische Schule)
- ☐ 2 **Obligatorische Schule** (8 oder 9 Jahre obligatorische Schule / Real-, Sekundar-, Bezirks-, Orientierungsschule, Pro-/ Untergymnasium, Sonderschule)
- ☐ 3 **Diplommittelschule oder Berufsvorbereitende Schule** (2- bis 3-jährige Ausbildung: Diplommittelschule DMS, Fachmittelschule FMS, Verkehrsschule oder ähnliche Ausbildung)
- ☐ 4 **Berufslehre, Vollzeit-Berufsschule** (2- bis 4-jährige Ausbildung: Berufslehre, berufliche Grundbildung mit eidg. Fähigkeitszeugnis (z.B. KV) oder Berufsattest (oder gleichwertig) sowie Anlehre)
- ☐ 5 **Maturitätsschule** (Gymnasiale Maturität / Berufs- oder Fach-Maturität)
- ☐ 6 **Lehrerseminar** (vorbereitende Ausbildung für Lehrkräfte von Kindergarten, Primarschule, Handarbeit, Hauswirtschaft)
- ☐ 7 **Höhere Fach- und Berufsausbildung** (Höhere Berufsbildung mit eidg. Fachausweis / höhere Fachprüfung mit eidg. Diplom oder Meisterdiplom oder gleichwertige Ausbildung)
- ☐ 8 **Höhere Fachschule** (Höhere Fachschule (HF) für Technik (bzw. Technikerschule TS) / HF für Wirtschaft (bzw. HKG) oder ähnliche höhere Fachschule (2 Jahre Voll- oder 3 Jahre Teilzeitstudium)
- ☐ 9 **Fachhochschule**
- ☐ 10 **Universität, Hochschule** (Bachelor - Universität, ETH, Pädagogische Hochschule) (Master / Lizentiat / Diplom / Staatsexamen / Postgrad / Doktorat / Habilitation Universität)

22. Religiosität

Als wie religiös würden Sie sich beschreiben? Bitte geben Sie eine Einschätzung auf einer Skala von 1 bis 5. 1 bedeutet «überhaupt nicht religiös» und 5 bedeutet «sehr religiös».

1<br>Überhaupt nicht religiös	2	3	4	5<br>Sehr religiös
☐	☐	☐	☐	☐

23. Politische Einstellung

Wenn es um die Einordnung politischer Standpunkte geht, spricht man oft von «links» und «rechts». Wie würden Sie Ihre eigene politische Einstellung einstufen, wenn 1 «sehr links» bedeutet und 7 «sehr rechts»?

- ☐ 1 Sehr links
- ☐ 2
- ☐ 3
- ☐ 4
- ☐ 5
- ☐ 6
- ☐ 7 Sehr rechts

Introduction

1. L'âge[1]

Quelle est votre année de naissance (par ex. 1985)? ___________

2. Le sexe

☐ 1 masculin
☐ 2 féminin

3. Lieu de résidence

Quel est le numéro postal d'acheminement de votre domicile actuel ? ___________

4. Intérêt pour des sujets d'actualité

Je vous lis maintenant différents thèmes. Veuillez s.v.p. me dire sur une échelle de 1 à 5 à quel point chaque sujet vous intéresse. 1 signifie „pas du tout" et 5 signifie „énormément".

	1 Pas du tout	2	3	4	5 Enormé-ment
Politique nationale et internationale	☐	☐	☐	☐	☐
Economie et finances	☐	☐	☐	☐	☐
Sport	☐	☐	☐	☐	☐
Science et recherche	☐	☐	☐	☐	☐
Situations humaines, accidents, criminalité, etc.	☐	☐	☐	☐	☐

[1] "Ne sait pas" (98) était une option de réponse pour toutes les questions.

5. Thèmes de recherche

Nous souhaitons connaître votre opinion concernant la science et la recherche. À votre avis, la science devrait-elle effectuer des recherches sur les thèmes suivants au cours des 15 prochaines années? Vous pouvez à nouveau répondre sur une échelle allant de 1 à 5. 1 signifiant «pas du tout» et 5 «enormément».

	1 Pas du tout	2	3	4	5 Enormé-ment
Climat et énergie	☐	☐	☐	☐	☐
Santé et alimentation	☐	☐	☐	☐	☐
Transport et mobilité	☐	☐	☐	☐	☐
Communication et numérisation	☐	☐	☐	☐	☐
Développement économique et marché du travail	☐	☐	☐	☐	☐
Immigration et intégration	☐	☐	☐	☐	☐

Utilisation des médias et comportement en matière d'information

6. La fréquence d'utilisation de l'information

A quelle fréquence êtes-vous en contact avec la science et la recherche par le biais des médias ?
Vous pouvez à nouveau me répondre sur une échelle de 1 à 5. 1 signifie „jamais“, 5 signifie „très fréquemment“.

6.1. Utilisation hors ligne des informations

	1 Jamais	2	3	4	5 Très souvent
Télévision, sans médiathèques en ligne	☐	☐	☐	☐	☐
Dont à la télévision suisse SRF	☐	☐	☐	☐	☐
Radio	☐	☐	☐	☐	☐
Dont à la radio suisse SRF	☐	☐	☐	☐	☐
Quotidiens, journaux hebdomadaires ou magazines imprimés	☐	☐	☐	☐	☐
Magazines scientifiques, tels que „Technologist» ou «Pour la Science»	☐	☐	☐	☐	☐
Sur Internet	☐	☐	☐	☐	☐

6.2 Utilisation en ligne de l'information

FILTRE: SI Internet est utilisé = 2-5

	1 Jamais	2	3	4	5 Très souvent
Sites Web ou applications des journaux ou des magazines	☐	☐	☐	☐	☐
Médiathèques de chaînes de télévision et de radio	☐	☐	☐	☐	☐
Pages web d’organismes scientifiques, d’autorités et d’organisations	☐	☐	☐	☐	☐
Facebook, Twitter ou autres réseaux sociaux	☐	☐	☐	☐	☐
Blogs ou forums en ligne	☐	☐	☐	☐	☐
Wikipédia	☐	☐	☐	☐	☐
YouTube ou plateformes vidéo similaires	☐	☐	☐	☐	☐

7. Réglages convexes: Communication sur les médias sociaux

FILTRE: SI Internet est utilisé = 2-5

Veuillez s.v.p. me dire à quelle fréquence vous faites ce qui suit par le biais d'Internet. 1 signifie „jamais", 5 signifie „très souvent".

	1 Jamais	2	3	4	5 Très souvent
Afficher ou partager des informations ou des opinions sur la science et la recherche	☐	☐	☐	☐	☐
Lier ou favoriser les informations ou les opinions sur la science et la recherche	☐	☐	☐	☐	☐
Commenter les informations ou les opinions sur la science et la recherche	☐	☐	☐	☐	☐

8. Attention à la science dans les médias

Nous avons encore quelques questions supplémentaires concernant les comptes-rendus sur la science et la recherche dans les médias. Avec quelle attention suivez-vous ces informations ? 1 signifie „pas du tout attentivement", 5 signifie „très attentivement".

1 Pas du tout attentivement	2	3	4	5 Très attentivement
☐	☐	☐	☐	☐

9. Satisfaction à l'égard de la couverture médiatique

Et au niveau tout à fait général, à quel point êtes-vous satisfait du compte rendu sur la science et la recherche ? 1 signifie „pas du tout satisfait", 5 signifie „très satisfait".

1 Pas du tout satisfait	2	3	4	5 Très satisfait
☐	☐	☐	☐	☐

10. L'évaluation de la couverture médiatique

Dans quelle mesure approuvez-vous les déclarations suivantes? Le compte rendu des médias sur la science et la recherche est en général …

	1 N'approuve pas du tout	2	3	4	5 Approuve totalement
Digne de confiance.	☐	☐	☐	☐	☐
compréhensible.	☐	☐	☐	☐	☐
détaillé.	☐	☐	☐	☐	☐
Présente la science et la recherche sous un aspect négatif.	☐	☐	☐	☐	☐

11. L'utilisation de sources d'information autres que les médias de masse

On peut entrer en contact avec la science et la recherche non seulement par les médias, mais également pas d'autres moyens. Je vous cite quelques activités. Veuillez me dire dans quelle mesure vous pratiquez ces activités. 1 signifie „jamais ", 5 signifie „très souvent".

	1 Jamais	2	3	4	5 Très souvent
Visiter des musées et des expositions qui ont la science et la recherche pour thèmes	☐	☐	☐	☐	☐
Visiter des parcs zoologiques, des aquariums ou des jardins botaniques	☐	☐	☐	☐	☐
Aller à des manifestations, écouter des exposés et des discussions sur la science et la recherche	☐	☐	☐	☐	☐
Regarder des films et des séries qui se rapportent aussi à la science et la recherche	☐	☐	☐	☐	☐
Discuter avec des amis et connaissances sur la science et la recherche	☐	☐	☐	☐	☐
Discuter dans des services de messagerie instantanée comme Whatsapp sur la science et la recherche	☐	☐	☐	☐	☐

12. Traitement heuristique et systématique de l'information et contrôle comportemental perçu

J’aimerais maintenant bien avoir votre avis sur la science et la recherche. 1 signifie „n’approuve pas du tout “ et 5 „approuve totalement “.

	1 N’approuve pas du tout	2	3	4	5 Approuve totalement
Je suis bien informé sur la science et la recherche	☐	☐	☐	☐	☐
Je cherche des informations sur la science et la recherche de façon ciblée	☐	☐	☐	☐	☐
Je suis très au courant de la science et la recherche.	☐	☐	☐	☐	☐
Il importe que l’on soit informé sur la science et la recherche.	☐	☐	☐	☐	☐

Perception de la science

13. Objectifs de la science

Il y a différentes opinions concernant la fonction de la science. Je vous lis quelques déclarations. Veuillez me dire à quel point vous les approuvez.

	1 N'approuve pas du tout	2	3	4	5 Approuve totalement
La recherche scientifique est nécessaire, même s'il n'en résulte aucun avantage immédiat.	☐	☐	☐	☐	☐
La recherche scientifique devrait être soutenue par l'Etat.	☐	☐	☐	☐	☐
Les scientifiques devraient informer le public de leurs activités.	☐	☐	☐	☐	☐
Les scientifiques devraient plus écouter ce que les gens ordinaires pensent.	☐	☐	☐	☐	☐
Les décisions politiques devraient s'appuyer sur des bases scientifiques.	☐	☐	☐	☐	☐
Les personnes comme moi devraient prendre part à la décision sur quels thèmes les scientifiques doivent faire des recherches.	☐	☐	☐	☐	☐
Je participerai une fois volontiers à des recherches sur des projets scientifiques.	☐	☐	☐	☐	☐
La science et la recherche jouent un rôle important dans ma vie.	☐	☐	☐	☐	☐

14. Reservations vs. beliefs in the promise of science

Qu'en pensez-vous : quelles sont les répercussions de la science et de la recherche sur notre vie ?

	1 N'approuve pas du tout	2	3	4	5 Approuve totalement
La science et la recherche peuvent résoudre chaque problème.	☐	☐	☐	☐	☐
La science et la recherche améliorent notre vie.	☐	☐	☐	☐	☐
La science et la recherche modifient notre mode de vie trop rapidement.	☐	☐	☐	☐	☐
L'avantage de la science et de la recherche est plus important que les éventuels dégâts qui pourraient survenir.	☐	☐	☐	☐	☐
La science devrait pouvoir tout explorer sans limitation.	☐	☐	☐	☐	☐
La science nous transmettra un jour une image complète du fonctionnement de la nature et de l'univers.	☐	☐	☐	☐	☐
Nous comptons trop sur la science.	☐	☐	☐	☐	☐

15. La confiance dans la science

Sur une échelle de 1 à 5, où 1 signifie „très faible“ et 5 „très élevé“, comment qualifieriez-vous le degré de votre confiance dans …

	1 Très faible	2	3	4	5 Très élevé
…les scientifiques au sein des Universités?	☐	☐	☐	☐	☐
…la science en général ?	☐	☐	☐	☐	☐

16. Confiance dans les scientifiques (METI)

Quelles sont les qualités que vous associez aux scientifiques en rapport avec leur recherche? Nous vous nommons une qualité et son contraire. Si vous croyez que cette qualité se rapporte fortement aux scientifiques, vous lui donnez un 5. Si vous pensez que cette qualité ne s'applique pas du tout aux scientifiques, attribuez un 1. Les chiffres intermédiaires vous permettent de nuancer votre jugement.

	1	2	3	4	5	
incompétent	☐	☐	☐	☐	☐	compétent
inexpérimenté	☐	☐	☐	☐	☐	expérimenté
non qualifié	☐	☐	☐	☐	☐	qualifié
hypocrite	☐	☐	☐	☐	☐	franc
malhonnête	☐	☐	☐	☐	☐	honnête
injuste	☐	☐	☐	☐	☐	juste
amoral	☐	☐	☐	☐	☐	moral
non éthique	☐	☐	☐	☐	☐	éthique
irresponsable	☐	☐	☐	☐	☐	responsable

17. Science Populism Scale

Il s’agit maintenant de la relation entre la science et la société. Dans quelle mesure êtes-vous d’accord avec les énoncés suivants?

	1<br>N`approuve pas du tout	2	3	4	5<br>Approuve totalement
Le peuple devrait avoir une influence sur le travail des scientifiques.	☐	☐	☐	☐	☐
En cas de doute, on devrait plutôt faire confiance à l’expérience des gens simples qu’aux estimations des scientifiques.	☐	☐	☐	☐	☐
Nous devrions nous baser davantage sur le bon sens commun et moins sur les études scientifiques.	☐	☐	☐	☐	☐
Les scientifiques ne voient que leur propre avantage.	☐	☐	☐	☐	☐
Les scientifiques sont de mèche avec la politique et l’économie.	☐	☐	☐	☐	☐
Les gens simples partagent des valeurs et des intérêts communs.	☐	☐	☐	☐	☐
Ce qui unit les gens simples, c’est qu’ils font confiance à leur bon sens dans la vie quotidienne.	☐	☐	☐	☐	☐
Les gens simples ont en commun un caractère bon et honnête.	☐	☐	☐	☐	☐
La science fait partie de l’élite de notre pays.	☐	☐	☐	☐	☐

18. Scientific Literacy

Je vous lis maintenant quelques déclarations que vous connaissez éventuellement de l’école ou des médias. Quelques-unes sont fausses et d’autres sont justes. Veuillez me dire si, à votre avis, ces déclarations sont justes ou fausses et si vous en êtes sûr. Si vous ne connaissez pas la réponse par rapport à une déclaration, dites-le-moi tout simplement. Nous passons alors à la déclaration suivante.

	1<br>Certaine ment faux	2<br>Plutôt faux	3<br>Plutôt juste	4<br>Certaine ment juste	98<br>Ne sait pas
Les continents sur lesquels nous vivons se déplacent depuis des millions d’années (juste)	☐	☐	☐	☐	☐
Les électrons sont plus petits que les atomes (juste)	☐	☐	☐	☐	☐
Les antibiotiques tuent aussi bien les virus que les bactéries (faux)	☐	☐	☐	☐	☐
Les gènes de la mère décident si un enfant sera un garçon ou une fille (faux)	☐	☐	☐	☐	☐
Les théories scientifiques ne changent jamais. (faux)	☐	☐	☐	☐	☐

19. Désenchantement des médias à l'égard du système

Indépendamment du thème de la science: que pensez-vous en général des médias en Suisse? Par médias, nous entendons des médias d’informations établis, qui informent sur les actualités. Veuillez indiquer si vous êtes plutôt d’accord ou non avec les énoncés suivants.

	1 N`approuve pas du tout	2	3	4	5 Approuve totalement
Je pense que le système des médias d’information est corrompu.	☐	☐	☐	☐	☐
Les médias d’information agissent avant tout dans leur propre intérêt.	☐	☐	☐	☐	☐
Les agissements des médias me rendent furieux/furieuse.	☐	☐	☐	☐	☐
Les médias d’information aident à résoudre les problèmes importants de la société.	☐	☐	☐	☐	☐

Variables de personnalité et sociodémographie

Pour terminer, voici encore quelques questions vous concernant personnellement.

20. Expérience directe avec la science

a. Etes-vous vous-même un ou une scientifique?

☐ 1 Oui
☐ 2 Non

Filtre: SEULEMENT si F20 a. = 2

	1<br>Oui	2<br>Non
Connaissez-vous un ou une scientifique?	☐	☐
Avez-vous professionnellement affaire avec un ou une scientifique?	☐	☐
Avez-vous des membres de la famille qui ont étudié ou étudient encore ?	☐	☐

21. L'éducation

Quelle est la formation que vous avez achevée en dernier ?

☐ 1 **Aucune scolarité** (aucune scolarité / école primaire, pas au-delà de la 7ème année scolaire)
☐ 2 **Scolarité obligatoire** (8 ou 9 ans / école secondaire, école d'arrondissement, cycle d'orientation, progymnase, école à enseignement spécial)
☐ 3 **école de degré diplôme ou école de préparation professionnelle** (Formation de la durée de 2 ou 3 ans: école de degré diplôme, école de culture générale ECG, école d'administration et des transports, ou formation équivalente)
☐ 4 **Apprentissage professionnel, école professionnelle à plein temps** (Formation de la durée de 2 à 4 ans: apprentissage professionnel, formation professionnelle initiale sanctionnée par un certificat fédéral de capacité ou par une attestation fédérale de formation ou formation équivalente et formation élémentaire)
☐ 5 **Maturité** (maturité / maturité professionnelle ou maturité spécialisée)
☐ 6 **école normale** (pour futurs enseignants des écoles enfantines, écoles primaires, travaux manuels, travaux ménagers)
☐ 7 **Formation professionnelle supérieure** (Formation professionnelle supérieure avec brevet fédéral ou examen professionnel supérieur avec diplôme fédéral ou maîtrise ou formation équivalente)
☐ 8 **écoles supérieures** (Ecoles supérieures techniques (ou école technique ET), écoles supérieures d'économie (ou ESCG) ou école professionnelle supérieure équivalente (2 ans à plein temps ou 3 ans à temps partiel)
☐ 9 **Haute école spécialisée**
☐ 10 **Université, haute école** (Bachelor - Université, EPF, haute école spécialisée, haute école pédagogique)
(Master / licence / diplôme / Examen d'Etat / postgrade / doctorat / habilitation universitaire)

22. La religiosité

A quel point vous considérez-vous religieux ? Veuillez donner une estimation sur une échelle de 1 à 5. 1 signifie „pas du tout religieux“ et 5 signifie „très religieux“.

1<br>Pas du tout religieux	2	3	4	5<br>Très religieux
☐	☐	☐	☐	☐

23. Attitude politique

Quand on parle de classification du point de vue politique, on dit volontiers „gauche“ et „droite“. Comment jugeriez-vous votre propre position au niveau politique, si 1 correspond à „très à gauche“ et 7 „très à droite“ ?

☐ 1 Très à gauche
☐ 2
☐ 3
☐ 4
☐ 5
☐ 6
☐ 7 Très à droite

Introduzione

1. L'età

In quale anno è nato/a (es. 1985)? ___________

2. Il sesso

☐ 1 Maschile
☐ 2 Femminile

3. Il luogo di residenza

Qual è l'NPA del suo attuale luogo di domicilio? ___________

4. Interesse per temi di attualità

Adesso Le leggerò alcuni temi. La prego di dirmi, su una scala da 1 a 5, quanto è interessato/a a ciascun tema. 1 significa "per niente" e 5 significa "moltissimo".

	1 Per niente	2	3	4	5 Moltissimo
Politica nazionale ed estera	☐	☐	☐	☐	☐
Economia e finanze	☐	☐	☐	☐	☐
Sport	☐	☐	☐	☐	☐
Scienza a ricerca	☐	☐	☐	☐	☐
Destini umani, casi sfortunati, crimini ecc.	☐	☐	☐	☐	☐

[1] "Non so" (98) era un'opzione di risposta a tutte le domande.

5. **Temi di ricerca**

Ora desideriamo porle alcune domande sulla sua opinione in merito alla scienza e alla ricerca. Cosa ne pensa? In che misura la scienza dovrebbe studiare i seguenti argomenti nei prossimi 15 anni? Può rispondere sempre con una scala da 1 a 5. 1 significa "per niente" e 5 significa "moltissimo".

	1 Per niente	2	3	4	5 Moltissimo
Clima ed energia	☐	☐	☐	☐	☐
Salute e nutrizione	☐	☐	☐	☐	☐
Trasporti e mobilità	☐	☐	☐	☐	☐
Comunicazione e digitalizzazione	☐	☐	☐	☐	☐
Sviluppo economico e mercato del lavoro	☐	☐	☐	☐	☐
Immigrazione e integrazione	☐	☐	☐	☐	☐

Uso dei media e comportamento informativo

6. Frequenza di utilizzo delle informazioni

Quanto spesso entra in contatto con la scienza e la ricerca grazie ai media?
Può rispondere di nuovo con una scala da 1 a 5. 1 significa "mai" e 5 significa "entro in contatto con la scienza e la ricerca molto spesso grazie a questo mezzo di comunicazione".

6.1. Uso offline delle informazioni

	1 Mai	2	3	4	5 Molto spesso
Televisione, escluse mediateche online	☐	☐	☐	☐	☐
tra cui la Televisione svizzera, SRF	☐	☐	☐	☐	☐
Radio	☐	☐	☐	☐	☐
tra cui la Radio svizzera, SRF	☐	☐	☐	☐	☐
Quotidiani stampati, settimanali o riviste	☐	☐	☐	☐	☐
Riviste scientifiche come „National Geographic“ o „Le Scienze“	☐	☐	☐	☐	☐
in Internet	☐	☐	☐	☐	☐

6.2. Uso delle informazioni online

FILTRO: SE Internet usato=2-5

Siti web o app di giornali e riviste	☐	☐	☐	☐	☐
Mediateche di emittenti televisive e radiofoniche	☐	☐	☐	☐	☐
Siti web di istituti, autorità e organizzazioni scientifiche	☐	☐	☐	☐	☐
Facebook, Twitter o altri social network	☐	☐	☐	☐	☐
Blog o forum online	☐	☐	☐	☐	☐
Wikipedia	☐	☐	☐	☐	☐
YouTube o altre piattaforme video	☐	☐	☐	☐	☐

7. Impostazioni convesse: Comunicazione sui social media

FILTRO: SE Internet usato=2-5

La prego di dirmi quanto spesso compie le seguenti azioni in Internet. 1 significa "mai" e 5 significa "molto spesso".

	1<br>Mai	2	3	4	5<br>Molto spesso
Postare o condividere informazioni o opinioni su scienza e ricerca	☐	☐	☐	☐	☐
Aggiungere "mi piace" o "preferito" a informazioni o opinioni su scienza e ricerca	☐	☐	☐	☐	☐
Commentare le informazioni o le opinioni su scienza e ricerca	☐	☐	☐	☐	☐

8. L'attenzione per la scienza nei media

Abbiamo qualche altra domanda sulle relazioni e i rapporti scientifici e di ricerca nei media. Quanto attentamente li segue? 1 significa "per niente attento/a" e 5 significa "molto attento/a".

1<br>Per niente attento/a	2	3	4	5<br>Molto attento/a
☐	☐	☐	☐	☐

9. Zufriedenheit mit Medienberichterstattung

In generale, quanto è soddisfatto/a degli articoli e dei servizi sulla scienza e la ricerca? 1 significa "per niente soddisfatto/a" e 5 significa "molto soddisfatto/a".

1<br>Per niente soddisfatto/a	2	3	4	5<br>Molto soddisfatto/a
☐	☐	☐	☐	☐

10. Valutazione della copertura mediatica

Quanto è d'accordo con le seguenti affermazioni? Gli articoli e i servizi dei media sulla scienza e la ricerca in generale...

	1<br>Non sono assolutamente d'accordo	2	3	4	5<br>Sono assolutamente d'accordo
affidabili	☐	☐	☐	☐	☐
comprensibili.	☐	☐	☐	☐	☐
completi.	☐	☐	☐	☐	☐
Rappresentano negativamente la scienza e la ricerca.	☐	☐	☐	☐	☐

11. Uso di fonti di informazione non provenienti da mezzi di comunicazione di massa

Si può entrare in contatto con la scienza e la ricerca non solo grazie ai media ma anche in altri modi. Le indico alcune attività. La prego di dirmi quanto spesso le svolge. 1 significa "mai" e 5 significa "molto spesso".

	1<br>Mai	2	3	4	5<br>Molto spesso
Visitare musei e mostre su scienza e ricerca	☐	☐	☐	☐	☐
Visitare zoo, acquari o giardini botanici	☐	☐	☐	☐	☐
Assistere a conferenze e discussioni su scienza e ricerca	☐	☐	☐	☐	☐
Guardare film e serie che trattano anche di scienza e ricerca	☐	☐	☐	☐	☐
Parlare di scienza e ricerca con amici e conoscenti	☐	☐	☐	☐	☐
Scambiare idee sulla scienza e la ricerca tramite app di messaggistica come WhatsApp	☐	☐	☐	☐	☐

12. **Elaborazione euristica e sistematica dell'informazione e controllo comportamentale percepito**

Adesso gradiremmo conoscere la Sua opinione sulla scienza e la ricerca. 1 significa "non sono assolutamente d'accordo" e 5 "sono assolutamente d'accordo".

	1<br>Non sono assolutamen te d'accordo	2	3	4	5<br>Sono assolutament e d'accordo
Sono ben informato/a su scienza e ricerca	☐	☐	☐	☐	☐
Cerco informazioni in modo mirato su scienza e ricerca	☐	☐	☐	☐	☐
So molte cose di scienza e ricerca	☐	☐	☐	☐	☐
È importante essere informati su scienza e ricerca.	☐	☐	☐	☐	☐

Percezione della scienza

13. Obiettivi della scienza

Ci sono varie opinioni su quali siano le funzioni della scienza. Adesso Le leggerò alcune affermazioni. La prego di dirmi quanto è d'accordo.

	1 Non sono assolutament e d'accordo	2	3	4	5 Sono assolutament e d'accordo
La ricerca scientifica è necessaria anche se non produce un'utilità immediata.	☐	☐	☐	☐	☐
La ricerca scientifica dovrebbe essere sostenuta dallo Stato.	☐	☐	☐	☐	☐
Gli scienziati dovrebbero informare la gente sul loro lavoro.	☐	☐	☐	☐	☐
Gli scienziati dovrebbero prestare più attenzione a quello che pensa la gente comune.	☐	☐	☐	☐	☐
Le decisioni politiche dovrebbero basarsi sulle conoscenze scientifiche.	☐	☐	☐	☐	☐
La gente come me dovrebbe partecipare alle decisioni sui temi oggetto di ricerca scientifica.	☐	☐	☐	☐	☐
Mi piacerebbe partecipare una volta a qualche progetto scientifico.	☐	☐	☐	☐	☐
La scienza e la ricerca svolgono un ruolo importante nella mia vita.	☐	☐	☐	☐	☐

14. Reservations vs. beliefs in the promise of science

Secondo Lei: quali ripercussioni hanno la scienza e la ricerca sulla nostra vita?

	1 Non sono assolutament e d'accordo	2	3	4	5 Sono assolutament e d'accordo
La scienza e la ricerca possono risolvere ogni problema.	☐	☐	☐	☐	☐
La scienza e la ricerca migliorano la nostra vita	☐	☐	☐	☐	☐
Con la scienza e la ricerca la nostra vita cambia troppo velocemente.	☐	☐	☐	☐	☐
L'utilità della scienza e della ricerca è maggiore delle possibili conseguenze negative.	☐	☐	☐	☐	☐
La scienza dovrebbe poter fare ricerca su tutto senza limitazioni.	☐	☐	☐	☐	☐
Un giorno la scienza ci fornirà un quadro completo del funzionamento della natura e dell'universo.	☐	☐	☐	☐	☐
Ci affidiamo troppo alla scienza.	☐	☐	☐	☐	☐

15. Fiducia nella scienza

Su una scala da 1 a 5, in cui 1 indica un livello "molto basso" e 5 un livello "molto alto", come valuta il Suo livello di fiducia …

	1 Molto basso	2	3	4	5 Molto alto
…negli scienziati nelle università?	☐	☐	☐	☐	☐
…nella scienza in generale?	☐	☐	☐	☐	☐

16. Fiducia negli scienziati (METI)

Cosa pensa degli scienziati rispetto alla loro ricerca? Le diremo una proprietà e il suo opposto. Se pensi che questa proprietà sia completamente calzante per gli scienziati, assegni un 5. Se pensi che questa proprietà non si applichi agli scienziati, premiate con un 1. Può graduare la sua opinione, utilizzando i valori intermedi.

	1	2	3	4	5	
incompetenti	☐	☐	☐	☐	☐	competenti
inesperti	☐	☐	☐	☐	☐	esperti
non qualificati	☐	☐	☐	☐	☐	qualificati
corrotti	☐	☐	☐	☐	☐	integri
disonesti	☐	☐	☐	☐	☐	onesti
ingiusti	☐	☐	☐	☐	☐	giusti
immorali	☐	☐	☐	☐	☐	morali
non etici	☐	☐	☐	☐	☐	etici
irresponsabili	☐	☐	☐	☐	☐	responsabili

17. Science Populism Scale

Ora parliamo del rapporto tra scienza e società. Quanto è d'accordo con le seguenti affermazioni?

	1<br>Non sono assolutament e d'accordo	2	3	4	5<br>Sono assolutament e d'accordo
Il popolo dovrebbe poter influire sul lavoro degli scienziati.	☐	☐	☐	☐	☐
In caso di dubbio, ci si dovrebbe fidare dell'esperienza di vita della gente comune piuttosto che delle stime degli scienziati.	☐	☐	☐	☐	☐
Dovremmo affidarci di più al buon senso e meno agli studi scientifici.	☐	☐	☐	☐	☐
Gli scienziati sono orientati solo al loro tornaconto personale.	☐	☐	☐	☐	☐
Gli scienziati fanno affari sottobanco con il mondo politico ed economico.	☐	☐	☐	☐	☐
La gente comune condivide gli stessi valori e interessi.	☐	☐	☐	☐	☐
Ciò che unisce la gente comune è la fiducia nel buon senso nella vita quotidiana.	☐	☐	☐	☐	☐
La gente comune è generalmente buona e onesta.	☐	☐	☐	☐	☐
La scienza fa parte dell'élite del nostro paese.	☐	☐	☐	☐	☐

18. Scientific Literacy

Ora le leggerò alcune affermazioni che probabilmente già conosce dalla scuola e dai media. Alcune sono errate, alcune esatte. La prego di dirci se secondo Lei queste affermazioni sono esatte o sbagliate e se ne è sicuro/a. Se non sa cosa rispondere per un'affermazione, lo dica tranquillamente. In tal caso passeremo all'affermazione successiva.

	1<br>Sicurame nte errata	2<br>Probabil mente errata	3<br>Probabil mente esatta	4<br>Sicurame nte esatta	98<br>Non so
I continenti su cui viviamo si muovono già da milioni di anni. (esatto)	☐	☐	☐	☐	☐
Gli elettroni sono più piccoli degli atomi (esatto)	☐	☐	☐	☐	☐
Gli antibiotici uccidono sia i virus sia i batteri. (errato)	☐	☐	☐	☐	☐
I geni della madre determinano se nascerà un maschio o una femmina. (errato)	☐	☐	☐	☐	☐
Le teorie scientifiche non cambiano mai. (errato)	☐	☐	☐	☐	☐

19. Disincanto dei media relativi al sistema

Indipendentemente dal tema scienza, cosa ne pensa in generale dei media in Svizzera? Per media si intendono i mezzi di informazione affermati che forniscono informazioni sugli eventi di attualità. Per favore, indichi quanto è d'accordo con ciascuna affermazione seguente.

	1 Non sono assolutamente d'accordo	2	3	4	5 Sono assolutamente d'accordo
Penso che il sistema dei mezzi di informazione sia corrotto.	☐	☐	☐	☐	☐
I mezzi di informazione agiscono principalmente nel proprio interesse.	☐	☐	☐	☐	☐
Quello che fanno i mezzi di informazione mi fa arrabbiare.	☐	☐	☐	☐	☐
I mezzi di informazione aiutano a risolvere gli importanti problemi della società.	☐	☐	☐	☐	☐

Variabili di personalità e sociodemografia

Per concludere l'intervista abbiamo ancora qualche domanda su di Lei.

20. Esperienza diretta con la scienza

a. Lei è uno/a scienziato/a?

☐ 1 Sì
☐ 2 No

Filtro: Se F20 a.=2

	1<br>Sì	2<br>No
Conosce personalmente uno/a scienziato/a?	☐	☐
Ha a che fare professionalmente con la scienza?	☐	☐
Ha familiari che hanno studiato o studiano?	☐	☐

21. Istruzione

Qual è l'ultima formazione da Lei conseguita?

☐ 1 **Nessuna formazione conclusa** (nessuna formazione/scuola elementare, fino a 7 anni di scuola dell'obbligo)
☐ 2 **Scuola dell'obbligo** (8 o 9 anni di scuola obbligatoria/scuola di grado secondario I con esigenze elementari o elevate, scuola distrettuale, scuola media d'orientamento, (pro)ginnasio, scuola speciale)
☐ 3 **Diploma di scuola media o scuola professionale** (formazione della durata di 2 o 3 anni: scuola media di diploma, scuola di cultura generale, scuola d'amministrazione o formazione equivalente)
☐ 4 **Tirocinio professionale, scuola professionale a tempo pieno** (formazione della durata da 2 a 4 anni: tirocinio professionale, formazione professionale di base con attestato federale di capacità o attestato federale di formazione pratica (o equivalente) e formazione empirica)
☐ 5 **Scuola di maturità** (maturità liceale/maturità professionale o specializzata)
☐ 6 **Istituto magistrale** (per la formazione degli insegnanti di scuola materna, scuola elementare, lavoro manuale, economia domestica)
☐ 7 **Formazione specialistica o professionale superiore** (formazione professionale superiore con attestato professionale federale/esame professionale superiore con diploma federale o maestria oppure formazione equivalente)
☐ 8 **Scuola professionale superiore** (scuola professionale superiore (HF), scuola per tecnici ST/HF für Wirtschaft (o HKG) o formazioni analoghe (2 anni a tempo pieno o 3 anni a tempo parziale)
☐ 9 **Scuola universitaria professionale**
☐ 10 **Università, scuola universitaria**

22. La religiosità

Quanto religioso/a si descriverebbe? La prego di rispondere in base ad una scala da 1 a 5. 1 significa "per niente religioso" e 5 significa "molto religioso".

1<br>Per niente religioso	2	3	4	5<br>Molto religioso
☐	☐	☐	☐	☐

23. Atteggiamento politico

L'orientamento politico è spesso definito di "sinistra" o di "destra". Come classificherebbe il Suo orientamento politico, tenendo presente che 1 significa "molto di sinistra" e 7 "molto di destra"?

☐ 1 Molto di sinistra
☐ 2
☐ 3
☐ 4
☐ 5
☐ 6
☐ 7 Molto di destra
