# Supplementary material for: Who supports science-related populism? A nationally representative survey on the prevalence and explanatory factors of populist attitudes toward science in Switzerland
Source: PLoS One. 2022 Aug 8;17(8):e0271204. doi: 10.1371/journal.pone.0271204 (PMC9359586; doi:10.1371/journal.pone.0271204)
Supplement: Attachment — Submitted filename: PLOS_SciPop-in-Switzerland_Rebuttal-Letter_REV02.docx [file pone.0271204.s004.docx]

**Response to Reviewer Comments on ms.
“Who supports science-related populism? A nationally representative survey on the prevalence and explanatory factors of populist attitudes toward science in Switzerland”

(2^nd^ revision)**

We thank the reviewers for their comments on the revised version of our manuscript. These were once more very helpful in improving the quality of the study, as they entailed worthwhile suggestions and considerations. Therefore, we were happy to follow (most of) them in a second revision, which included the following changes:

- Following the suggestion by reviewer 1, we re-ran all hypothesis tests with trimmed survey weights.
- Following another suggestion by reviewer 1, we now explain the reasons for our decision to model trust in science/scientists as an explanatory factor of science-related populism and not vice versa, and discuss implications of this decision.
- Responding to comments by reviewer 2, we ran additional analyses that used a Bollenian instead of the Goertzian SciPop Score.
- We also followed all other minor suggestions by R1 and R2.
- In addition, we shortened some paragraphs to keep the article as concise as possible, and also adjusted the title.

Below we explain the changes in detail and respond to further comments. We hope that the editor and reviewers will think that our revision has further improved the paper so that it is suitable for publication.

**Comments of Reviewer 1 (R1) and Authors’ Responses**

***R1****: I wish to thank the authors for the good work they have carried out so far and their complete and polite replies to my comments. I think that the article has clearly improved and we are close to a definitive and publishable version.*

**AUTHORS**: Thank you very much! We appreciated your comments on the first version of the manuscript, which motivated this improvement.

***R1****: I still have, however, two doubts: 1. Weights: to the best of my knowledge, it is commonly considered a best practice to trim excessive weight to avoid the possible effects of outlying weighted observations. A commonly applied trimming policy is at a around 5 or 7 max (and usually 1/5 or 1/7 as a min weight). Having 0.04 as min and close to 24 as max does rise some concerns on the possible effect of outlying weighted observations. As such, I would add, as a robustness check, a rerun of Table 2 with trimmed weights.*

**AUTHORS**: This is a good point, thank you for bringing it up. You are completely right: Having extreme variance in the weights can introduce bias into the analyses, so we tested if outliers affected the analyses and re-ran them with trimmed weights.

When trimming the weights, we decided against general rules of thumb that consider all weights smaller than 1/7 or 1/5 and bigger than 5 or 7 extreme, for example. Instead, we used a sample-specific trimming range, which accounts for the characteristic variance of weights in our data set and can therefore be considered a more reliable approach to weight trimming (see Kerckhove et all., 2014). To determine that range, we applied the inter-quartile range method, which relies on the median of the weights and the 25%-75% quartile range of the weights. With this method, we specified a trimming range of 1/4.56 (= 0.22) and 4.56 (for more details, see Supplementary Information, p. 5)

After trimming our weights to this range, we repeated the analyses for Table 2. The results – which we present in Supplementary Table A11 – differed marginally from the results of the original analyses, as we now point out in the manuscript:

We then trimmed the weights to this interval, re-ran the hypothesis tests—and found that the results differed only very marginally from those of the analyses with untrimmed weights (see Table A11 in Appendix S1). The direction of all coefficients remained the same, their size changed only slightly, and none of the explanatory variables lost or reached significance, except for two: Residing in Italian-speaking Switzerland did not link to science-related populist attitudes anymore, whereas trust in scientists did.

We then discuss these results in the last paragraph of the results section:

Overall, the results of these two sensitivity tests support those of the original analyses and substantiate their robustness. Moreover, they indicate that science-related populism might also link to lower trust in scientists (H3d). At the same time, they suggest that the association between science-related populist attitudes and interest in science is less pronounced in case the non-compensatoriness premise of science-related populism is ignored (H3a), and that the association between science-related populist attitudes and residence place may be confounded by our sampling design (H1f). These observations should be further explored in future research.

To account for the uncertainty as to whether trust in scientists and region of residence are significant explanatory factors of science-related populism, we added references to the trimmed analyses in the first part of the results section…

Science-related populists are less likely to […] live in the Italian-speaking region of Switzerland (H1f; *but see additional analyses below*). […] However, we do not find that science-related populism and trust in science or scientists have a relationship that is independent of the sociodemographic and attitudinal covariates included in the model (H3c and H3d; *but see additional analyses below*).

…and discussed the discrepancies these analyses unearthed in the discussion section:

But some of these findings require closer scrutiny: Sensitivity tests showed that people’s interest in science and place of residence might not be as closely linked to science-related populism as the main analyses indicated, whereas trust in scientists could indeed be. Future studies should therefore try to replicate our own […].

***R1:*** *2. Trust in science/scientists as independent variables: This is more a suggestion than a request like the one before. There is a certain tendency in contemporary literature to, let me be blunt, run exploratory analyses with inferential models (such as the OLS). I understand that the authors do not claim causality, still in a multivariate OLS you are testing a model where you expect a certain directionality. This is actually still present in the text: these are your explanatory variables (and it is clearly expressed in the text, in the table and in the R code). Why do expect these Xs to explain your dependent variable? What is the theoretical assumption behind the fact that you decided to put them on the right side instead of the left side of the equation? Do not get me wrong: this changes nothing to the rest of the article, but you are not just showing a correlation matrix identifying the significant corr between variables. By using a regression you are testing a series of hypotheses, in this case of an addictive effect of trust in science/scientists keeping all other covariates equal. You cannot claim causality and say "this is the correct directionality", but you still can and should say why, theoretically, you think this is the proper way to represent and test this relationship. While true in general, in this case given the clear conceptual overlap between the constructs it would be better to clarify the authors' reasoning: just adding covariates because they turned out to be related/significant in other studies is not enough. A short paragraph, given the large amount of other variables that have been tested, would suffice and improve the overall quality of the text, while at the same time recognizing a limit and setting up new research (that I would gladly read by the way).*

**AUTHORS**: Thank you for sharing these worthwhile considerations, which had not been detailed in the manuscript yet—although they do indeed deserve further elaboration. Therefore, we added two paragraphs that explain why we treated trust in science/scientists as explanatory variables of science-related populist attitudes (and not vice versa), and what caveats (and potentials for future research) this brings about:

First, we revised a paragraph in the Literature Review section, now arguing why trust in science/scientists may be conceived as an explanatory factor of science-related populism and could be put on the right side of the regression equation:

[Scholarship] assumed that trust can reduce negative attitudes toward science, because trustworthiness perceptions partly derive from a rather stable “propensity to trust” [21] that can attenuate more volatile reservations against science—such as science-related populist attitudes, which may fluctuate considerably over time [70]. However, the reverse causality is also conceivable, as trust can be as volatile as science-related populist attitudes and may therefore be reduced by them [53,65,72].Empirical research offers little evidence on the direction of the relationship between trust in science and populist attitudes toward it, but it does indicate that such a relationship exists: […]

Second, we added some sentences to the Discussion section. These point out that our analytical approach is indeed limited in that it treats all covariates as constants when explaining science-related populist attitudes with trust in science/scientists. Moreover, we suggest that further research should address this limitation:

[Future studies] should also scrutinize the association between science-related populism and trust in science or scientists: We modelled trust as an antecedent of populism, drawing on existing research that conceived trust as a rather stable disposition, which may shape more volatile orientations, such as sciences-related populist attitudes [70]. Accordingly, our analyses tested how much variance in science-related populist attitudes can be explained by trust when other covariates remain constant. But science-related populism might also be an antecedent of trust in science [35], so future studies should also test how much variance in trust can be explained by science-related populism.

**Comments of Reviewer 2 (R2) and Authors’ Responses**

***R2****: I thank the authors for improving the quality of the paper after this first step of revision. Unfortunately, I still have a serious concern about the computation of the science-related populism score. Less importantly, the specificity of science-related populism should be better underlined (or dimmed).*

*– About the "goertzian" vs. "bollenian" approaches of computing the populism score, the explanations of the authors lend me to think that, contrary to their intention, the goertzian appears to me as a bad response to a good question. The justification of the authors for choosing the goertzian approach is the following :*

*[explanation from the Appendix S1]*

*I am not convinced by this justification. If the authors fear that some individuals could endorse some aspects and reject other aspects of scientific populism, and that scientific populism should be non-compensatory, my opinion is that they solve this problem by the goertzian approach by adding more serious problems. In regression analyses, the authors will predict a score that is not similar across participants! As the score does not reflect the same items, I really do think that the regression will be statistically flawed (because some of the predictors of the same analysis will predict some items for some participants, and some other items for other participants).*

*The crucial question whether subdimensions are correlated or not may be in my view more classically and properly (I am not opposed of course by new approaches and analyses, but not if they seem to unclarify the analyses, as it appears to me here) addressed by correlational analyses (Cronbach's alpha, and/or oblique PCA, and/or model comparisons of 1-factor vs. 4-factors solutions by CFA). Perhaps the simplest and closest to the existing results way of doing this is to perform a regression on the overall average score with a sufficient Cronbach alpha, and then performing distinct regressions for each subscore, as still presented in table 2 (so only changing the goertzian score by a bollenian score in the overall regression).*

*I would really (and I guess other scholars also will) be more confident about the results of the regression on the classical average score (Bollen), rather than on the goertzian score.*

*I would also add that the authors used the bollenian approach when computing the four subdimensions of populism (average of the two items) and not the goertzian approach (lower score of the two items), which is not consistent. But the computation of the goertzian score may be kept for comparison with other studies (in table 1).*

***AUTHORS****:* Thank you for sharing your critique with us. We understand that you have concerns about our approach to measure science-related populist attitudes. After all, similar concerns are currently debated in research on political populism as well. However, we are confident that the Goertz approach is both theoretically defensible and methodologically sound—but we nevertheless probed your concerns in additional analyses that used the Bollenian instead of Goertzian SciPop Scores and that are now included in the paper.

In our understanding, your main concern is that our regression analyses may not predict the same phenomenon for different respondents, because the SciPop Score is based on different items for different respondents. While we understand your concern, we do not share the rationale behind it. Similar to Wuttke et al. [22], we think that the SciPop Score does indeed reflect the same items for all respondents, because it always entails information on all eight items of the SciPop Scale (aside from missing values). We would like to cite Wuttke et al. (2020, p. 361) here: “Using the minimum may appear statistically inefficient at first as this operationalization seems to disregard the information on all but the lowest attribute. However, as the mathematical representation of the minimum function shows (see further below), the computation of the minimum, in fact, does consider all attributes when identifying the attribute with the lowest value.”

Accordingly, a Goertzian SciPop Score does consider all items of the SciPop Scale, otherwise it would be unable to determine the overall level of science-related populism. As such, it reflects responses to all eight items—like the Bollen approach—albeit it processes these responses differently. Therefore, both Goertzian and Bollenian regressions will predict the same items for all respondents.

So considering that the Goertz approach is theoretically and methodologically defensible, and given that this approach—and very similar regression models like ours—are frequently applied in political populism research [76–78], we think that it is sensible to keep the main analysis and results in their current form.

However, we nevertheless tested whether we would have obtained different results if we had not applied the Goertz approach to compute the SciPop Score but relied on the Bollen approach. Therefore, we repeated all hypothesis tests using Bollenian SciPop Scores instead of Goertzian SciPop Scores. The results are presented in Table A10 in Appendix S1, and we also describe and discuss them in the manuscript:

Bollenian analyses yielded results that did not differ considerably from those of the original analyses: Science-related populist attitudes were still negatively associated with university education, proximity to science, and scientific literacy, for example (see Table A10 in Appendix S1). Many associations were even stronger than in the Goertzian analyses, which is plausible because Bollenian SciPop Scores tend to be less conservative estimates of science-related populist attitudes than Goertzian SciPop Scores. As a consequence, some explanatory factors showed significant correlations with science-related populism, although they had not done so in the Goertzian analyses (e.g., trust in scientists and religiosity). Meanwhile, Bollenian analyses did not offer clear evidence that science-related populism is less widespread in Italian-speaking Switzerland and more widespread among people with high interest in science, even if the original analyses had showed this (see Table 2).

We go on to point out:

Overall, the results of these two sensitivity tests support those of the original analyses and substantiate their robustness. […] the same time, they suggest that the association between science-related populist attitudes and interest in science is less pronounced in case the non-compensatoriness premise of science-related populism is ignored (H3a) […]. These observations should be further explored in future research.

Meanwhile, we are also confident that the Bollen approach is most useful to quantify the four components of science-related populist attitudes: first, because these components do not represent non-compensatory concepts, and second, because this procedure is common practice in research on political populism as well, as we point out in the manuscript:

However, the four theoretical components of science-related populism themselves do not represent non-compensatory concepts. This means that they must be operationalized—most usefully—with the Bollen approach, which is common practice in current populism research [79–81]. Accordingly, we used respondents’ average agreement with the four subscales of the SciPop Scale to quantify conceptions of the ordinary people, conceptions of the academic elite, demands for decision-making sovereignty, and demands for truth-speaking sovereignty […].

***R2:*** *About my remark on the specificity of science-related populism, I am not fully satisfied by the response of the authors. I would remove the term "specific" and let only "variant" in the summary and along the article, as I still advocated for. For me, replacing "distinct" by "specific" is only some kind of rephrasing. Similarly, the sentence p. 5 "Such anti-scientific ideas represent a specific variant of populism, which differs conceptually from political populism—and has been conceptualized as “science-related populism” [6]." is not satisfying to me. Either the authors should explain what is exactly specific with regard to other forms of populism, or they should only write ""Such anti-scientific ideas represent a variant of populism, which has been conceptualized as “science-related populism” [6].". These changes will be more consistent with the conclusion of the authors that "further studies measuring both science-related and political populism must investigate this finding—and could then also test the conceptual assumption that the former and the latter are distinct populism variants".*

**AUTHORS**: We understand your concern about the distinctiveness of science-related populism. This is perhaps something that deserves a more detailed discussion (albeit we believe that Mede & Schäfer, 2020, provide reasonable arguments about why science-related populism is indeed conceptually distinct from political populism). Such discussion goes beyond the scope of the present study, so we followed your suggestion and removed the term “specific,” e.g., in the abstract and the Conceptual Framework section).

***R2:*** *In p. 17, I would propose to say that there is a slight tendency to have more science-related populism on the right that on the left, because all betas are positive, the overall beta is very close to significance, and one beta is significant.*

**AUTHORS**: We followed this suggestion—but still emphasize that science-related populism may have a slight tendency to occur on the right and center of the political spectrum, which is an important nuance in our findings:

This suggests that science-related populism has a slight tendency to occur on the right and center of the political spectrum—and tends to be less pronounced on the left, which supports H2a.

***R2:*** *P. 18, I would precise what groups the authors refer to when saying: "While demands for truth-speaking sovereignty appear to be raised by very specific population groups of the population".*

**AUTHORS**: Thanks for this suggestion, which we followed:

[D]emands for truth-speaking sovereignty appear to be raised by people who are older, do not hold a university degree, have little proximity to science, and have low trust in scientists (Adj. R2 = 0.30; F (df) = 16.76 (14, 908), p < 0.001; see Table 2).

**R2**: PP. 19-20, all p-values could be rounded at 3 decimals

***AUTHORS****: Done.*

**R2**: P. 24, I would say that Didier Raoult is an "isolated expert" rather than "pseudo-expert", as he was a renowned expert before covid-19 (and incidentally, you will avoid to be fired on French social media !!!).

***AUTHORS****: Thank you for this good hint. We changed the formulation according to your suggestion:*

[…] qualitative studies may explore how science-related populist attitudes are acquired during socialization [105], triggered by key events [18], and driven by conspiracy theorists like Daniele Ganser in Switzerland or alternative experts like Didier Raoult in France [102].
